# The prevalence of mental health-related multimorbidity during pregnancy: a systematic review and meta-analysis

**DOI:** 10.1186/s12889-026-26545-8

**Published:** 2026-02-07

**Authors:** Adhithi Sreenivasan, Mary Hewitt, Veronika Tirado, Soha El-Halabi, Walter Osika, Claudia Hanson, Sunjuri Sun

**Affiliations:** 1 Department of Global Public Health, Karolinska Institutet, Stockholm, Sweden; 2 Swedish Centre for Impacts of Climate Extremes (climes), Karolinska Institutet, Stockholm, Sweden; 3 Aging Research Center & Center for Social Sustainability, Department of Neurobiology, Care Sciences and Society, Karolinska Institutet, Stockholm, Sweden; 4 London School of Hygiene and Tropical Medicine, London, United Kingdom

**Keywords:** Pregnancy, Multimorbidity, Mental health, Prevalence.

## Abstract

**Introduction:**

Multimorbidity during pregnancy, defined as the co-occurrence of two or more physical or psychological conditions, is an emerging global health concern associated with adverse birth outcomes. This systematic review addresses gaps in research by determining the global pooled prevalence of mental health-related multimorbidity amongst pregnant women and identifying key populations at higher risk of this type of multimorbidity.

**Methods:**

This study adhered to Preferred Reporting Items for Systematic Reviews and Meta-Analyses (PRISMA) guidelines and was pre-registered with PROSPERO (ID: CRD420251023056). A systematic search was conducted across MEDLINE, EMBASE, and Web of Science for peer-reviewed articles from January 1, 2015 to February 10, 2025, to capture the current landscape of mental health-related multimorbidity during pregnancy. Two independent reviewers screened titles and abstracts, full texts, extracted data, and assessed study quality using Covidence software. A proportional random-effects meta-analysis was conducted to calculate the global pooled prevalence of mental health-related multimorbidity during pregnancy.

**Results:**

The original search across databases yielded 5,989 studies. The global pooled prevalence of mental-health related multimorbidity during pregnancy across the eligible 92 studies representing over 357 million pregnant women was 1.90% (95% CI 1.73%-2.07%). The subgroup analysis by geographic region revealed that the prevalence estimate was lowest for Oceania at 0.36% (95% CI: 0.34%-0.37%) while it was the highest for Asia at 6.82% (95% CI: 5.37%-8.43%).

**Conclusion:**

Mental health-related multimorbidity during pregnancy is an under-researched issue in global health. Pregnant women would benefit from further studies, development of relevant policy, and greater awareness amongst public health and medical professionals to address needs.

**Supplementary Information:**

The online version contains supplementary material available at 10.1186/s12889-026-26545-8.

## Background

Multimorbidity during pregnancy is defined as the presence of two or more conditions during pregnancy, which may encompass both physical and mental health conditions [1]. Multimorbidity amongst adults is associated with poorer health outcomes, such as mortality and increased healthcare usage with findings showing a higher prevalence of multimorbidity in female adults compared to male adults, however multimorbidity amongst pregnant women specifically is still relatively underexplored [2]. Given gender variations in the prevalence of mental health conditions[3], it is important to investigate how the impacts of multimorbidity and mental health manifest in women, especially during a critical and dynamic period like pregnancy. There are major physiological changes that occur to support the demands of the growing foetus, such as the alterations of major organ systems like the cardiovascular and gastrointestinal systems, changes in hormone levels and endocrine output, shifts in immunological function, and reprioritisation of metabolism [4, 5].

 Both psychological and physical multimorbidity during pregnancy are associated with adverse birth outcomes[6], such as preterm birth, large and small weights for gestational age which are above the 90th and below the 10th percentiles respectively, and macrosomia [7]. Mental health conditions specifically have been found to be a risk factor for preterm birth and low birth weight [8]. According to the World Health Organization (WHO), 10% of pregnant women have a mental health disorder, most commonly depression, and this figure is higher in low-and middle-income (LMIC) nations, where it is estimated that 15.6% of the pregnant population have one or more mental health conditions [9]. Other studies report an even higher prevalence, with one review suggesting that upwards of 20% of pregnant women are impacted by perinatal mental illness and possibly are underdiagnosed [10].

Depression and anxiety during pregnancy are of particular concern since they are some of the most common mental health disorders that pregnant women experience [11]. These two conditions specifically are associated with shorter gestation, adverse foetal neurodevelopment, and lower birth weight [12]. Systematic reviews investigating the prevalence of antenatal depression in any form and anxiety reported that the proportion of pregnant women with depression was 20.7% and those with a clinical diagnosis of anxiety were 15.2% of the pregnant population [13]. Other mental health conditions of note among the pregnant population include schizophrenia, bipolar affective disorder, eating disorders, personality disorders, and suicidal ideation [13, 14]. Furthermore, evidence suggests that the presence of physical multimorbidity, such as diabetes, hypertension, asthma, and epilepsy, is associated with the emergence and persistence of mental health conditions [15, 16].

This study aimed to determine the global pooled prevalence of mental health-related multimorbidity during pregnancy, where at least one of the conditions were a mental health condition. Whilst previous systematic reviews have examined the prevalence of multiple high-risk factors during pregnancy[17], no other systematic reviews have reported on the prevalence of mental health-related multimorbidity during pregnancy to date. Thus, this review fills an important gap in the literature on mental health and multimorbid conditions during pregnancy.

## Methods

### Search strategy

This systematic review and meta-analysis investigates the prevalence of mental health-related multimorbidity amongst pregnant women globally. This systematic review was pre-registered with PROSPERO (CRD420250652064) including a protocol and followed the Preferred Reporting Items for Systematic Reviews and Meta-Analyses (PRISMA) reporting guidelines [18]. The PRISMA checklist for this review is available in Supplemental Table 1. With the purpose of securing a broad search for articles relevant to the topic of multimorbidity during pregnancy, a systematic literature search was performed in MEDLINE, EMBASE, and Web of Science in consultation with a specialist librarian. Advanced search options were used to filter within the databases from January 1, 2015 to February 10, 2025 for original peer-reviewed literature reporting on the prevalence of mental health and multimorbidity during pregnancy. As this systematic review focuses on contemporary evidence from the last decade and earlier studies may be less relevant to current practice, January 1, 2015 was chosen as the starting date for the search. Using the condition, context, population (CoCoPop) framework recommended by the Joanna Briggs Institute (JBI) for systematic reviews of prevalence[19], the condition was mental health-related multimorbidity, the context included any setting, and the population was pregnant women. There were no language restrictions. Mental health conditions considered eligible aligned with those found in the DSM-5. Comorbidities could be physical, psychological, and social in origin and had the potential to affect the health of the expectant mother and fetus. The following MeSH terms were used: *Pregnancy*, *Pregnant Women*, *Multiple Chronic Conditions*, *Multimorbidity*, *Comorbidity*, and *Prevalence*. Extended search strategies for each database are displayed in Supplemental Table 2.

### Eligibility criteria

Studies were eligible during the title and abstract screening as well as the full-text screening stages if they included pregnant women—from the first trimester until birth—who experienced mental health-related multimorbidity. Mental health-related multimorbidity was defined as having at least two or more conditions, where at least one condition was a mental health condition. Mental health conditions were defined as any condition outlined in the Diagnostic and Statistical Manual of Mental Disorders, Fifth Edition, Text Revision (DSM-5-TR), which included, for instance, schizophrenia spectrum and other psychotic disorders, bipolar and related disorders, personality disorders, substance-related and addictive disorders, depressive disorders, and anxiety disorders. Studies were included if (1) they reported on a mental health-related multimorbidity; (2) were observational studies (such as cohort or cross-sectional studies); and (3) were published since January 1 st, 2015.

As this review focused on including published articles that had undergone peer review, grey literature was not included in the search. Studies were excluded if they were animal studies or conference abstracts. Studies with an index condition were also excluded, for instance, studies that only reported on multimorbidity during pregnancy among those with asthma. Finally, studies were excluded if they did not include pregnant women as the primary population of interest, such as those that focus on non-pregnant women, children, or men, or if it was not possible to disaggregate data for pregnant women.

### Screening, data extraction and risk of bias assessment

Two reviewers independently screened the titles and abstracts of studies against the eligibility criteria. Full-text papers were sought for all studies that appeared to meet the eligibility criteria or for studies where the title and abstract did not provide sufficient evidence to reach a decision. The full-text papers were independently reviewed by two reviewers. Discrepancies were resolved by consensus between the two reviewers, with input from a third reviewer if required.

For all studies that met the eligibility criteria, the following data items were extracted: author names, date of publication, World Bank income region classification, continental region, country, study period, study population, study design, data source, ascertainment of morbidities, sample size, mean/median age in years, conditions, number of conditions included in definition, number of pregnant women with mental health-related multimorbidity, and prevalence of mental health-related multimorbidity during pregnancy. Only prevalence at baseline was extracted if reported at multiple time points.

The mental health-related multimorbidity that was most common was recorded for the prevalence calculation and served as the numerator. The number of pregnant women in the overall sample in each study was also recorded. The study characteristics and corresponding descriptions of the different mental health conditions involved in multimorbidity during pregnancy are available in Supplemental Table 3. This table provides a comprehensive view of the combinations of physical, psychological, and social conditions affecting pregnant women who experience at least one mental health condition.

An adapted version of the Newcastle-Ottawa Scale (NOS) was utilised concurrently while extracting data to assess the risk of bias for each study, which has been previously used for systematic reviews of prevalence [2, 20]. Three domains were included: selection, comparability, and outcomes. Two independent reviewers filled out the NOS form during data extraction and resolved any discrepancies through consensus.

### Certainty assessment and small study effects

The GRADE approach was utilised for the certainty assessment of the quality of the collective evidence presented in this systematic review [21]. The factors considered in this approach included risk of bias, inconsistency, indirectness, imprecision, publication bias. Furthermore, Egger’s test was conducted to specifically evaluate small study effects as they relate to the possibility of publication bias [22]. It must be noted that both approaches are limited in the extent to which they are applicable to systematic reviews of prevalence [23]. Supplemental Table 4 displays a summary of the GRADE approach applied to this review and Supplemental Fig. 1 shows the funnel plot for the body of literature included.

**Fig. 1:**
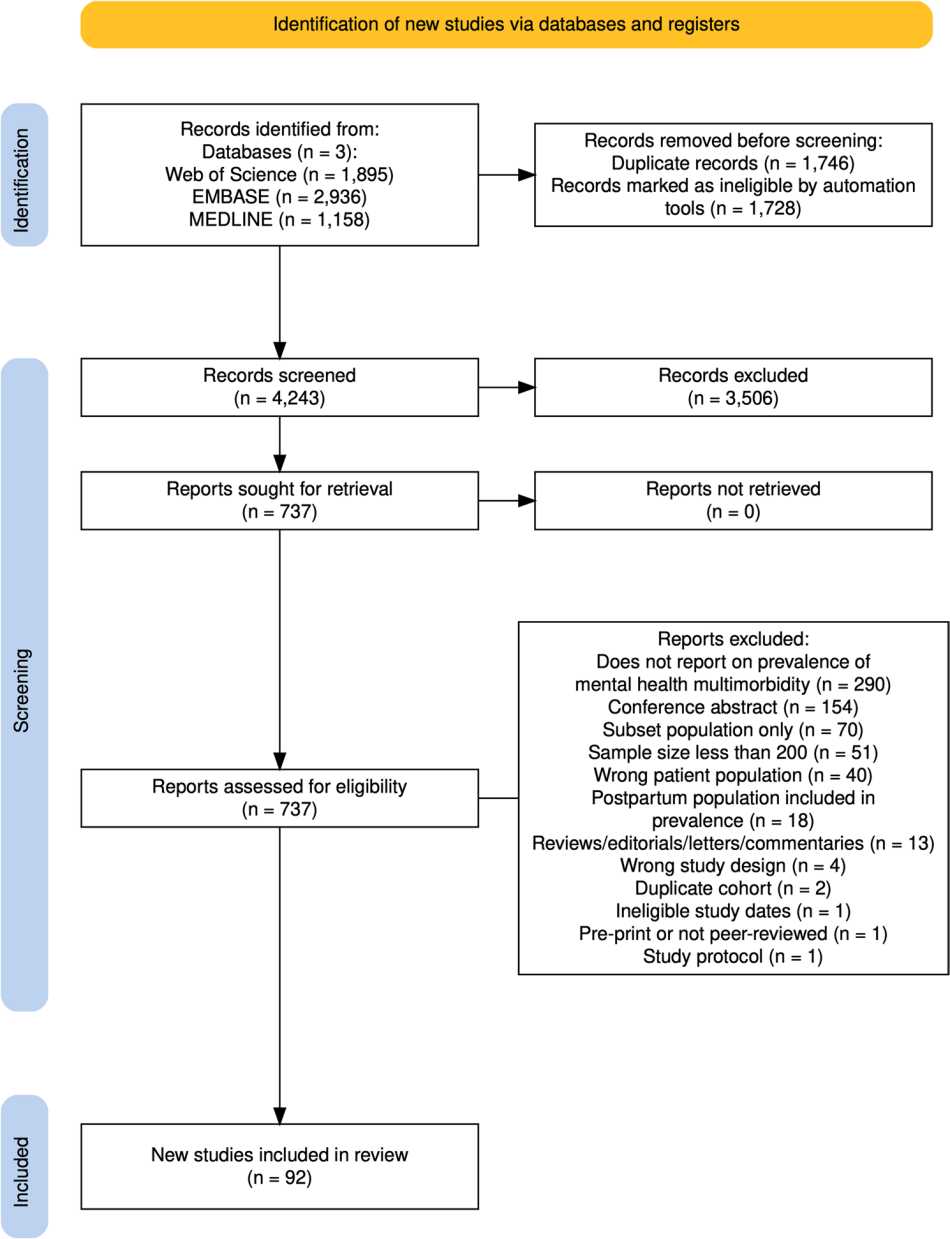
PRISMA flowchart

### Handling of missing data

Where there was missing or ambiguous data for the papers included, the study authors were contacted for clarification. Study authors were contacted at least twice via email. One study author was contacted regarding missing data on study years and a response was received. Two study authors were contacted regarding unclear reporting of the prevalence of mental health-related multimorbidity and one author responded with relevant data for the prevalence. The other author did not reply and their study was not included within the review.

### Data synthesis and meta-analysis

Data was aggregated and reported upon from each study in a table that was derived from a data extraction form used in Covidence. A proportional random effects meta-analysis was thus conducted using STATA 19 statistical software version using the “metaprop” package [24, 25]. The random-effects model was estimated using the DerSimonian-Laird method and the Freeman-Tukey double arcsine transformation was used to stabilise the within-study variances of these proportions [26]. Finally, individual prevalences for mental health-related multimorbidity during pregnancy were represented in a forest plot, along with the pooled prevalence. Subgroup analyses included geographical regions (i.e. continents), World Bank income region, presence of depression in multimorbidity during pregnancy, study design, ascertainment of morbidities (self-reported or objective), number of conditions included in multimorbidity during pregnancy definition, and multimorbidity comprised of only mental health-related conditions.

### Patient and public involvement

Patients or the public were not involved in the design, or conduct, or reporting, or dissemination plans of this systematic review.

## Results

### Identification of studies for systematic review

The initial search across the three databases (Web of Science, EMBASE, and MEDLINE) retrieved a total of 5,989 potential studies for screening. These references were uploaded to Covidence, where through automatic duplicate removal, 1,728 articles were removed from the pool of studies to be screened. Throughout the title and abstract screening, as well as during the full-text screening process, additional duplicates were removed manually. Studies were assessed against the eligibility criteria for this systematic review, leading to 737 articles that were screened in the full-text review stage. After screening these articles, a final number of 92 studies were included in this systematic review and meta-analysis [27–117]. Across all these studies, there were 357,383,705 (or over 357 million) pregnant women represented cumulatively. The smallest sample included was 282 pregnant women [101] whereas the largest sample size was 70,038,267 pregnant women [40]. The PRISMA flowchart in Fig. 1 shows the selection process throughout each screening stage [118]. 

### Characteristics of studies included

The majority of studies (*n* = 74, 80.43%) were conducted in high income countries (HICs), followed by upper-middle income countries (*n* = 16, 17.39%). Amongst the studies included, 45.65% were conducted in North America (*n* = 42), followed by 25% in Europe (*n* = 23) and 14.13% in Asia (*n* = 13). Two articles assessed multimorbidity during pregnancy in multi-continental and multinational contexts. Although each region is not necessarily represented in equal numbers within the review, there is representation from all geographic regions and World Bank income regions [119]. Additionally, all studies extracted were observational studies, of which 36 had a cross-sectional study design and 56 had a cohort study design.

### Assessment of risk of bias

According to the NOS scoring, 10 studies (10.87%) were of a lower quality, 34 studies (36.96%) were of a moderate quality, and 48 studies (52.17%) were of a high quality. Thus, the majority of the studies included within this systematic review were determined to have a low risk of bias. For further information on how each of the studies were scored during the quality assessment stage, Supplemental Table 5 shows the expanded NOS scoring for each of the nine questions included within the form.

### Meta-analysis of prevalence and meta-regression

The results from the meta-analysis indicated that the estimate of the global pooled prevalence of mental health-related multimorbidity among pregnant women is 1.90%, (95% CI, 1.73% to 2.07%).

**Fig. 2:**
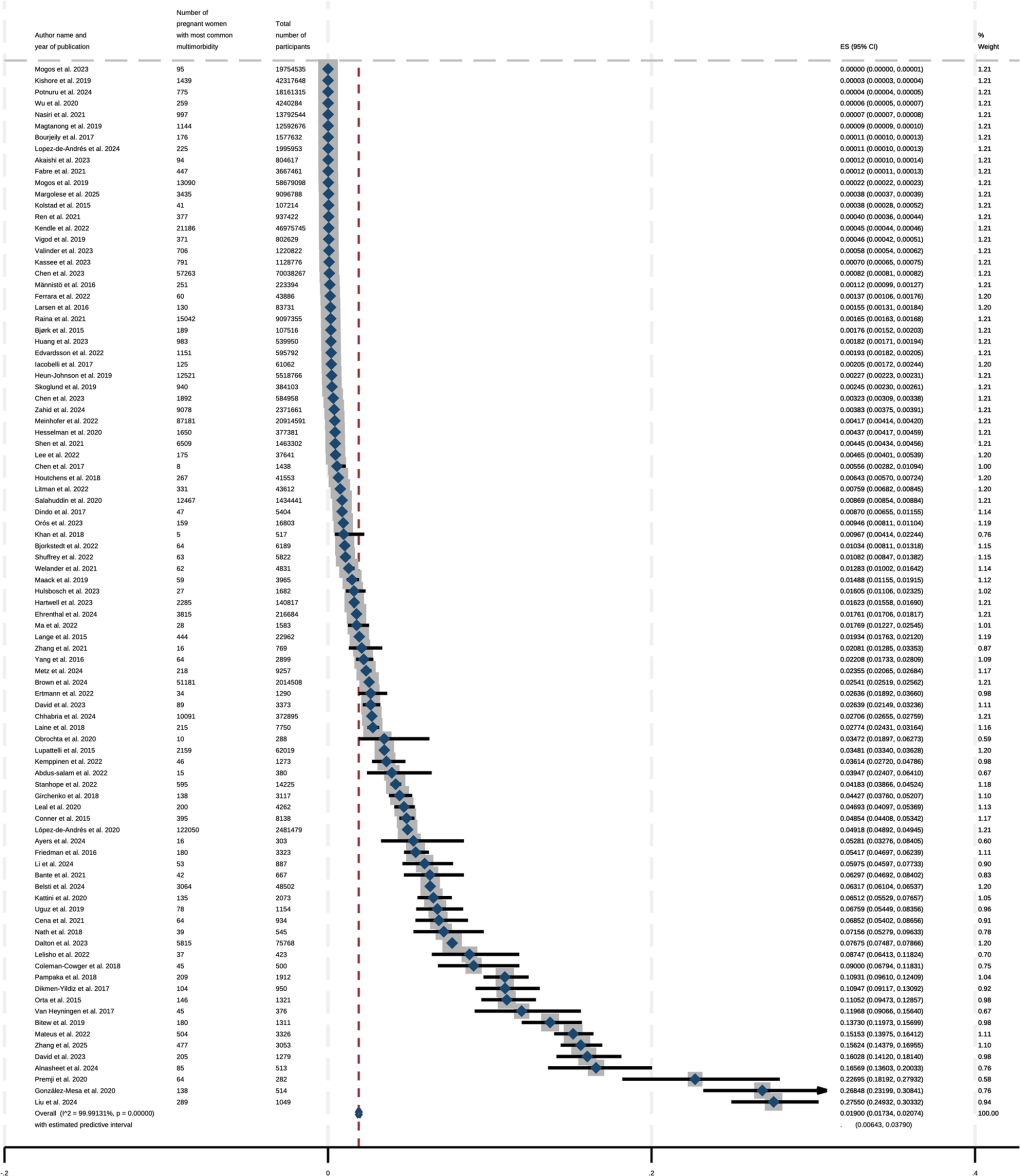
Overall prevalence of mental health-related multimorbidity during pregnancy

A forest plot depicting these results from the meta-analysis is represented in Fig. 2. None of the 92 studies included within this systematic review and meta-analysis reported a prevalence of mental health-related multimorbidity during pregnancy that was 0%.

**Fig. 3:**
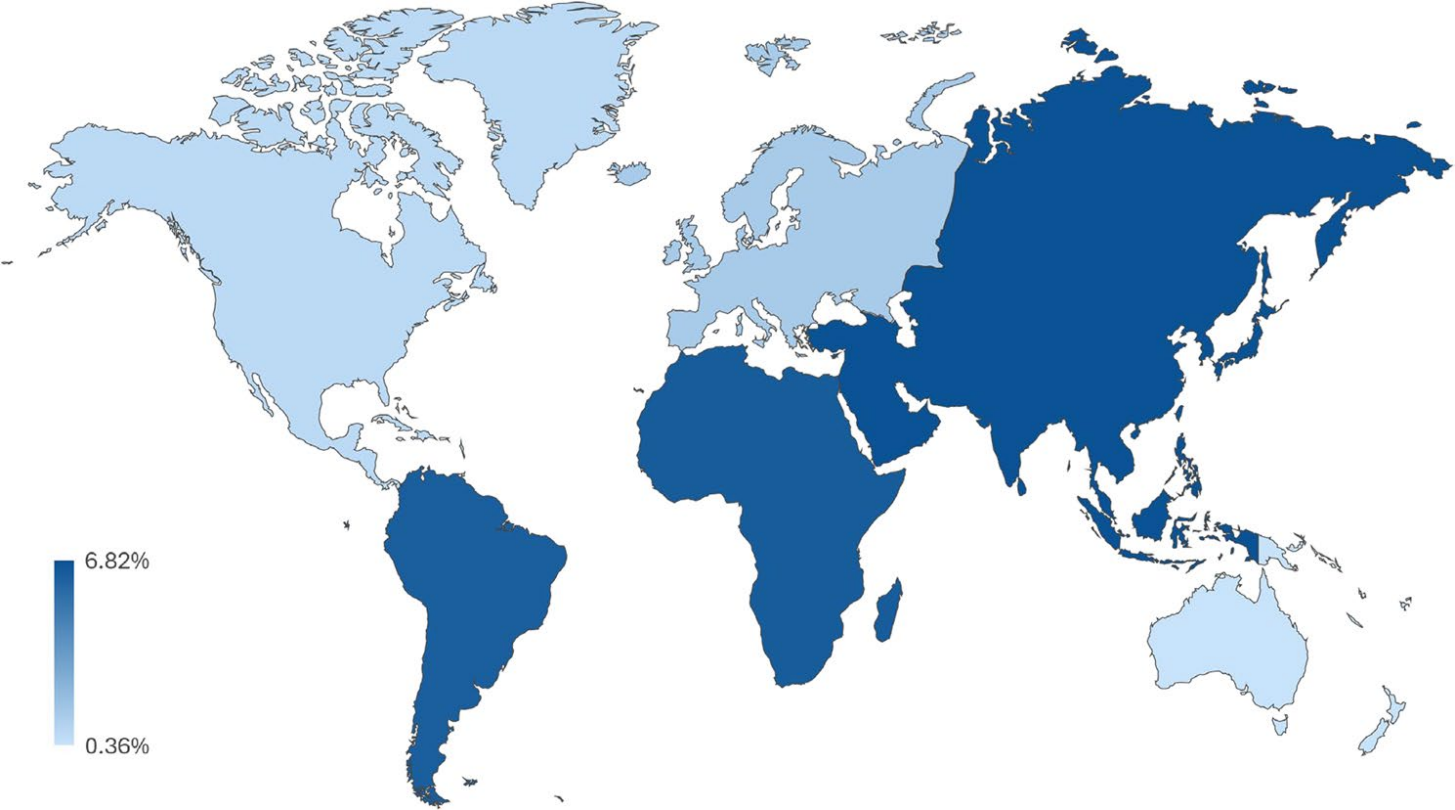
Visual map of mental health-related multimorbidity prevalence by continental region

**Fig. 4:**
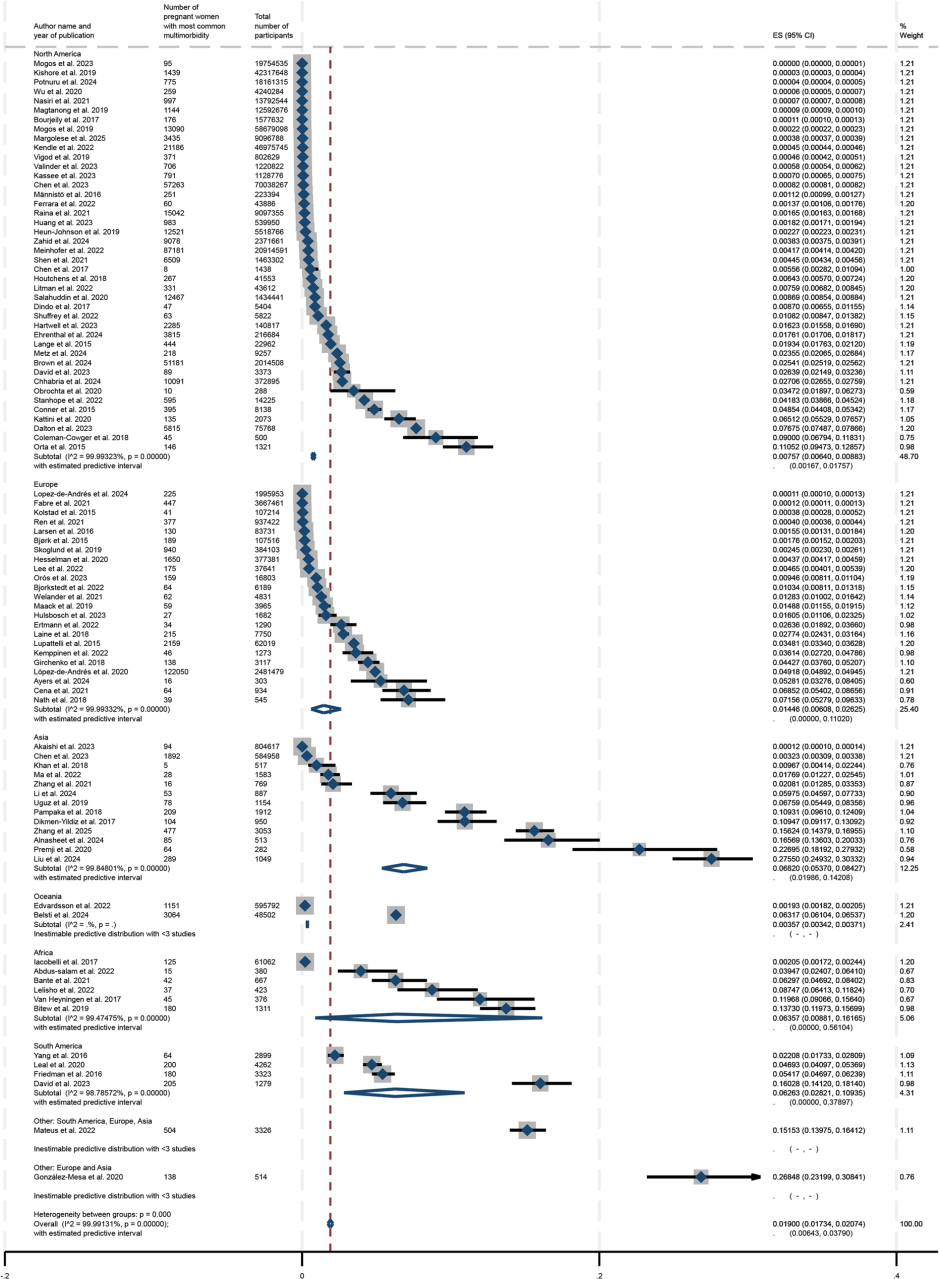
Subgroup analysis by continental region

**Fig. 5:**
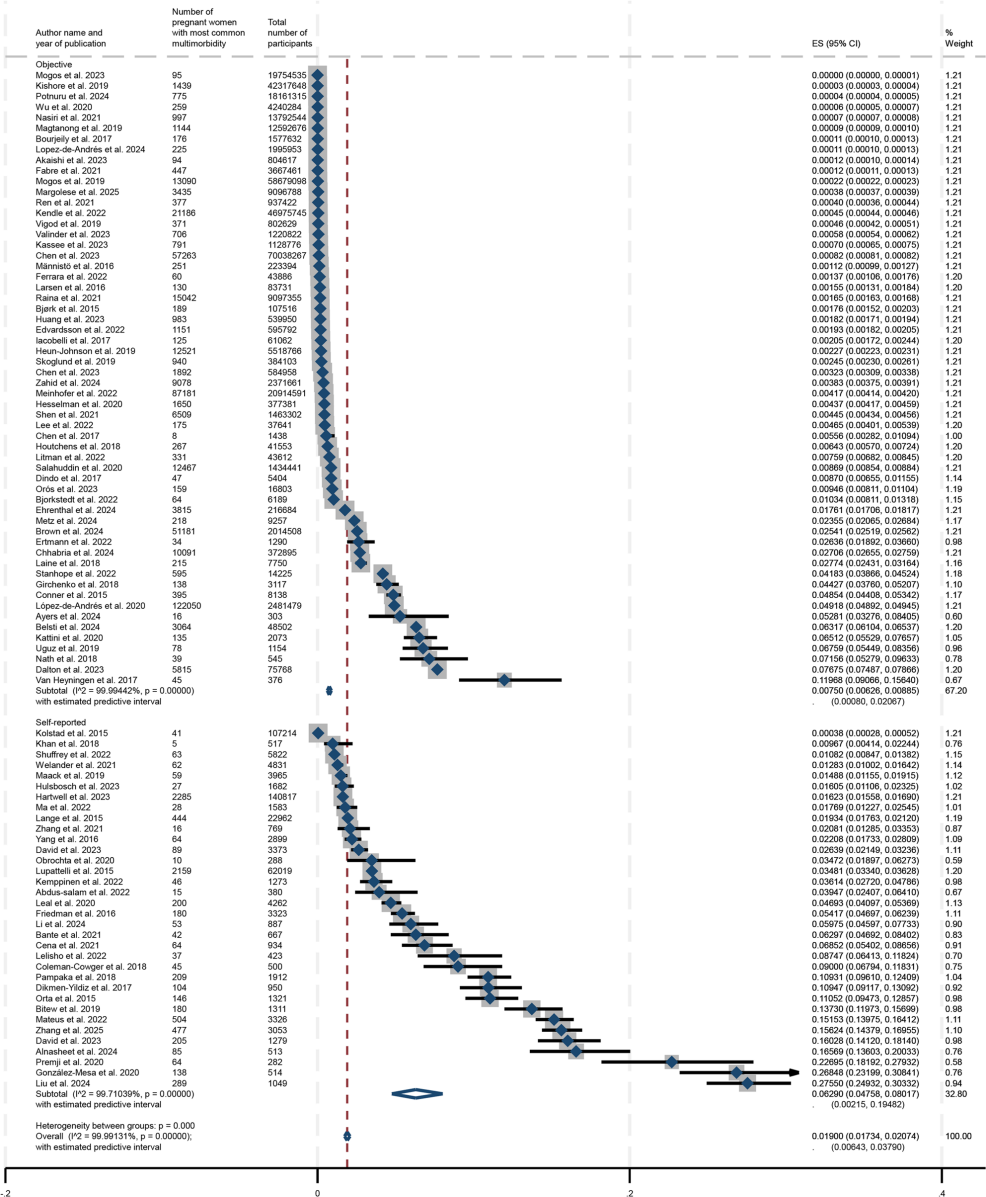
Subgroup analysis by ascertainment of morbidities

The respective forest plots for subgroup analyses by continental region and ascertainment of morbidities are represented in Figs. 3 and 4 and show the estimated prevalences for each subgroup as well as corresponding confidence intervals for these estimates. Additional subgroup analyses were conducted by study design (cohort or cross-sectional), presence of depression in the reported mental health-related multimorbidity during pregnancy, World Bank income, the number of conditions included in the definition of multimorbidity, as well as by mental health-only multimorbidity. These forest plots are available in Supplemental Figs. 2–6. In regard to the subgroup analysis by geographic region (shown in Fig. 5), the prevalence estimate was lowest for Oceania at 0.36% (95% CI: 0.34%−0.37%) while it was the highest for Asia at 6.82% (95% CI: 5.37%−8.43%). The prevalences for North America, Europe, South America, and Africa respectively were 0.76% (95% CI: 0.64%−0.88%), 1.45% (95% CI: 0.61%−2.63%), 6.26% (95% CI: 2.82%−10.94%) and 6.36% (95% CI: 0.88%−16.17%). The subgroup analysis by ascertainment of morbidities indicated that the prevalence for objective reporting was 0.75% (95% CI: 0.63%−0.89%) compared to self-reported mental health-related multimorbidity at 6.29% (95% CI: 4.76%−8.01%). Studies in which depression was involved during pregnancy had a higher prevalence of multimorbidity at 2.70% (95% CI: 2.43%−2.98%) compared to 1.26% (95% CI: 1.05%−1.49%) for studies that did not.

The meta-regression revealed that continental region, World Bank region, and ascertainment of morbidities represented the main sources of heterogeneity. Respectively, each factor explained 50.56%, 60.61%, and 40.21% of the heterogeneity between studies. The results of the meta-regression are in Supplemental Table 6. Furthermore, a sensitivity analysis was conducted and excluded the 10 studies that were assessed as having a higher risk of bias by the NOS scoring in this study. The analysis generated a global prevalence of mental-health related multimorbidity during pregnancy of 1.50% (95% CI, 1.34% to 1.66%). The forest plot for the sensitivity analysis can be found in Supplemental Fig. 7.

## Discussion

This is the first systematic review to date that has investigated the global pooled prevalence of mental health-related multimorbidity amongst pregnant women and to have recorded the various multimorbid mental health conditions across the existing literature. The global pooled prevalence from the meta-analysis of the studies included in this systematic review yielded an estimate of 1.90% of pregnant women experiencing mental health-related multimorbidity. Subgroup analyses displayed variations in the estimated prevalence of mental health-related multimorbidity during pregnancy, indicating that the lowest prevalence was in Oceania and the highest in Asia. Although the most common mental health conditions reported in studies were depression and anxiety, there was diversity in the types of mental health conditions experienced during pregnancy.

### Challenges in reporting mental health-related multimorbidity

The definition of multimorbidity is not broadly agreed upon, which contributes to a lack of standardisation in research about this topic [37]. Such uncertainties lead to individual researchers defining multimorbidity and associated characteristics differently, which can further exacerbate issues with reporting prevalence. Additionally, there is a need for diagnostic and screening technologies that have the capability to identify conditions that contribute to multimorbidity, particularly in lower-middle income countries, that are also not cost-prohibitive and are quick and simple to use [6]. Thus, it can be postulated that part of the reason for the unequal representation of income regions within this review is due to disparities in availability of necessary diagnostic tools. Furthermore, beyond the lack of standardisation in definitions of multimorbidity, there is no single multimorbidity index relevant to all healthcare settings [120]. The lack of a more exhaustive and widely applicable index has meant that the definition of multimorbidity used in the study may be derived from counting the conditions [76]. Such indices in tandem with an established definition of multimorbidity within the scientific community would introduce more standardisation and nuance into conversations regarding multimorbidity during pregnancy in the context of mental health. Variation in cut-off scores in even common data collection tools, like the Edinburgh Postnatal Depression Scale (EPDS), or the usage of different measures for one condition, like anxiety, complicate standardisation as well. Such tools applied in various settings are subject to different researchers’ interpretations of which score cut-offs constitute a risk of depression [121]. 

### Differences between clinical diagnosis and self-report measures

Although self-report measures of mental health are supported in the medical community, there is a consensus that they are not a replacement for traditional diagnostic evaluation by a trained professional, but rather a precursor to a clinical diagnosis [122, 123]. Ultimately, these screening instruments are meant to identify symptoms, and an individual’s perception of their own mental health symptoms is subjective. Additionally, questions on such self-report measures may be subject to misinterpretation, which would likely be addressed in a clinical setting or through a diagnostic interview. Beyond symptomology, following the ‘gold standard’ involves validating a self-report measure of mental health symptoms with a diagnostic interview [124]. It may be challenging for an individual to discern on a self-report questionnaire between mood changes due to hormonal fluctuations during pregnancy and indicators of more serious mental health issues like antenatal depression, for example [125]. However, it must be acknowledged that patient self-report measures are beneficial in capturing women’s lived experiences which is valuable in assessing mental health [126]. 

### Stigmatisation of mental health

It has been postulated that individuals in low- and middle-income countries experience a higher burden of perinatal mental health conditions [127]. Part of the burden may be influenced by stigmatisation of mental health. In a study investigating mental health stigma among pregnant women in Vietnam, 43.5% of pregnant women reported trying to hide their mental health issues, with stigmatising language towards mental health, shame, and low mental health literacy during pregnancy among the main reasons driving this concealment [128]. [129] Mental health during pregnancy is a difficult topic to broach in high income countries as well. In the United States, depression and anxiety are among the most underdiagnosed obstetric complications, as over 400,000 infants are born to mothers who are depressed each year [130, 131]. Therefore, stigmatisation of mental health conditions may have led to underreporting of mental health conditions during pregnancy.

### Identifying multimorbidity in multiple healthcare settings

Primary care may be able to capture a wider range of comorbidities beyond hospitalisations and emergency care for pregnant women. Mental health conditions experienced by pregnant women may be under-evaluated in hospital settings because conditions like anxiety and depression may be present in a more mild or moderate form before one becomes hospitalised [76]. Therefore, pregnant women who do not have severe mental health conditions may not be included in determinations of the prevalence of multimorbidity when hospitalisation records are used as a main source of information. Thus, primary care data sets may provide more comprehensive medical information and shed more light on less severe mental health conditions. Primary care physicians also commonly evaluate factors like smoking and alcohol use, which can be indicative of mental health issues during pregnancy and is information that may not necessarily be recorded in other medical settings [132]. 

However, primary care quality varies globally, and disparities between nations can mean that one nation with a strong primary care system may identify more multimorbidity among pregnant women, while another nation that suffers from lack of quality primary care training among clinicians may not be well suited to evaluate the complex nature of multimorbidity [133]. Another area for consideration is the fragmentation throughout the entire pipeline of medical care, which necessitates a greater integration of care [134]. The ability to follow pregnant women in a more cohesive manner through a coordinated system of care would allow for the identification of multimorbidity as it arises and, importantly, to address these conditions in a timely manner [135]. 

### Strengths and limitations of this systematic review

Methodologically, this study has several strengths. Each phase of screening was conducted through dual review with independent reviewers engaging in title and abstract screening, full-text screening, data extraction, and risk of bias assessment. In each of these stages, there was a consensus process in which both reviewers discussed the articles or data extraction results and came to an agreement. Few published studies expand on the variety of mental health-related multimorbidities during pregnancy, but this review reported on the various multimorbidities described across 92 studies and provided an estimate of the global pooled prevalence of mental-health related multimorbidity during pregnancy. This study also adds a dimension to existing literature on multimorbidity during pregnancy because it includes mental health conditions that are comorbid with social morbidities, such as intimate partner violence, and conditions that are understudied during pregnancy yet pose significant risk to the health of both the expectant mother and the foetus, such as eating disorders [136, 137].

The primary methodological limitation that affected the estimate of the pooled prevalence of this systematic review and meta-analysis was the lack of standardised reporting of multimorbidity during pregnancy, particularly relating to mental health. The prevalence of mental health-related multimorbidity amongst pregnant women was often dependent on the conditions reported in the study. For instance, a study reporting on depression amongst pregnant women would report a higher prevalence than studies that focused on rarer conditions, such as schizophrenia. Furthermore, the review consisted of a majority of studies from upper-middle and high income countries, leaving 5 studies that were conducted in lower-middle and low income countries[27]. Therefore, the generalisability of the estimated prevalence for these settings may be limited.

### Implications for global health research and policy

This study suggests there needs to be improved standardisation of the definition, reporting, and measurement of mental-health related multimorbidity. There is evidence that there are pregnant women throughout the world with multiple conditions and mental health issues, however there is a possibility that the true prevalence of mental health-related multimorbidity amongst pregnant women is underreported. However, these women are often excluded in current global health research because (a) there is still much unknown about how pregnant women deal with multimorbidity and (b) the concept of multimorbidity itself is still relatively new. Furthermore, the increasing prevalence of mental health conditions [138] and the complex interplay of biology and sociocultural factors involved [139, 140] deem mental health an important priority in research about multimorbidity during pregnancy. Thus, this study serves as a call for further global health research that sheds light on the burden of disease on pregnant women and the ways in which multimorbidity and mental health affect both themselves and their future children.

## Conclusions

Our estimate that 1.90% of women faced a multimorbidity with at least one condition related to mental health highlights a significant burden. The known complexity of managing multimorbidity, the appreciation of unique needs in pregnancy, together with the substantial burden, calls for targeted policies and strategies to support women’s health and wellbeing.

## Supplementary Information

Supplementary Material 1.

## Data Availability

This systematic review extracted data from previously published articles. Extracted data are available from the corresponding author upon reasonable request and are summarised in the article and supplementary materials.

## Abbreviations

EPDS: Edinburgh Postnatal Depression Scale
GRADE: Grading of Recommendations Assessment, Development and Evaluation
HICs: High-Income Countries
LMIC: Low- and Middle-Income Countries
NOS: Newcastle-Ottawa Scale
PRISMA: Preferred Reporting Items for Systematic reviews and Meta-Analyses
WHO: World Health Organization
