# Supplementary material for: The prevalence of mental health-related multimorbidity during pregnancy: a systematic review and meta-analysis
Source: BMC Public Health. 2026 Feb 7;26:714. doi: 10.1186/s12889-026-26545-8 (PMC12930893; doi:10.1186/s12889-026-26545-8)

Supplemental Table 1: PRISMA 2020 Main Checklist and Abstract Checklist

Topic	No.	Item	Location where item is reported
**TITLE**			
**Title**	1	Identify the report as a systematic review.	Page 1
**ABSTRACT**			
**Abstract**	2	See the PRISMA 2020 for Abstracts checklist	Page 2
**INTRODUCTION**			
**Rationale**	3	Describe the rationale for the review in the context of existing knowledge.	Introduction, page 5
**Objectives**	4	Provide an explicit statement of the objective(s) or question(s) the review addresses.	Introduction, page 5
**METHODS**			
**Eligibility criteria**	5	Specify the inclusion and exclusion criteria for the review and how studies were grouped for the syntheses.	Methods, page 5
**Information sources**	6	Specify all databases, registers, websites, organisations, reference lists and other sources searched or consulted to identify studies. Specify the date when each source was last searched or consulted.	Methods, page 5
**Search strategy**	7	Present the full search strategies for all databases, registers and websites, including any filters and limits used.	Supplemental Materials
**Selection process**	8	Specify the methods used to decide whether a study met the inclusion criteria of the review, including how many reviewers screened each record and each report retrieved, whether they worked independently, and if applicable, details of automation tools used in the process.	Methods, page 6
**Data collection process**	9	Specify the methods used to collect data from reports, including how many reviewers collected data from each report, whether they worked independently, any processes for obtaining or confirming data from study investigators, and if applicable, details of automation tools used in the process.	Methods, page 6

Topic	No.	Item	Location where item is reported
**Data items**	10a	List and define all outcomes for which data were sought. Specify whether all results that were compatible with each outcome domain in each study were sought (e.g. for all measures, time points, analyses), and if not, the methods used to decide which results to collect.	Methods, page 6
	10b	List and define all other variables for which data were sought (e.g. participant and intervention characteristics, funding sources). Describe any assumptions made about any missing or unclear information.	Methods, pages 6-7
**Study risk of bias assessment**	11	Specify the methods used to assess risk of bias in the included studies, including details of the tool(s) used, how many reviewers assessed each study and whether they worked independently, and if applicable, details of automation tools used in the process.	Methods, page 6
**Effect measures**	12	Specify for each outcome the effect measure(s) (e.g. risk ratio, mean difference) used in the synthesis or presentation of results.	Methods, page 6
**Synthesis methods**	13a	Describe the processes used to decide which studies were eligible for each synthesis (e.g. tabulating the study intervention characteristics and comparing against the planned groups for each synthesis (item 5)).	Methods, page 6
	13b	Describe any methods required to prepare the data for presentation or synthesis, such as handling of missing summary statistics, or data conversions.	Methods, page 7
	13c	Describe any methods used to tabulate or visually display results of individual studies and syntheses.	Methods, page 6
	13d	Describe any methods used to synthesize results and provide a rationale for the choice(s). If meta-analysis was performed, describe the model(s), method(s) to identify the presence and extent of statistical heterogeneity, and software package(s) used.	Methods, page 7
	13e	Describe any methods used to explore possible causes of heterogeneity among study results (e.g. subgroup analysis, meta-regression).	Methods, page 8
	13f	Describe any sensitivity analyses conducted to assess robustness of the synthesized results.	Methods, page 7
**Reporting bias assessment**	14	Describe any methods used to assess risk of bias due to missing results in a synthesis (arising from reporting biases).	Not applicable
**Certainty assessment**	15	Describe any methods used to assess certainty (or confidence) in the body of evidence for an outcome.	Methods, page 7

Topic	No.	Item	Location where item is reported
**RESULTS**			
**Study selection**	16a	Describe the results of the search and selection process, from the number of records identified in the search to the number of studies included in the review, ideally using a flow diagram.	Results, page 8
	16b	Cite studies that might appear to meet the inclusion criteria, but which were excluded, and explain why they were excluded.	Results, Figure 1
**Study characteristics**	17	Cite each included study and present its characteristics.	Results, page 8 and Supplemental Materials
**Risk of bias in studies**	18	Present assessments of risk of bias for each included study.	Results, page 9 and Supplemental Materials
**Results of individual studies**	19	For all outcomes, present, for each study: (a) summary statistics for each group (where appropriate) and (b) an effect estimate and its precision (e.g. confidence/credible interval), ideally using structured tables or plots.	Results, page 9 and Supplemental Materials
**Results of syntheses**	20a	For each synthesis, briefly summarise the characteristics and risk of bias among contributing studies.	Results, page 9
	20b	Present results of all statistical syntheses conducted. If meta-analysis was done, present for each the summary estimate and its precision (e.g. confidence/credible interval) and measures of statistical heterogeneity. If comparing groups, describe the direction of the effect.	Results, page 9 and Supplemental Materials
	20c	Present results of all investigations of possible causes of heterogeneity among study results.	Results, page 9
	20d	Present results of all sensitivity analyses conducted to assess the robustness of the synthesized results.	Results, page 9 and Supplemental Materials
**Reporting biases**	21	Present assessments of risk of bias due to missing results (arising from reporting biases) for each synthesis assessed.	Not applicable
**Certainty of evidence**	22	Present assessments of certainty (or confidence) in the body of evidence for each outcome assessed.	Supplemental Materials

Topic	No.	Item	Location where item is reported
**DISCUSSION**			
**Discussion**	23a	Provide a general interpretation of the results in the context of other evidence.	Discussion, page 10
	23b	Discuss any limitations of the evidence included in the review.	Discussion, page 12-13
	23c	Discuss any limitations of the review processes used.	Discussion, page 12-13
	23d	Discuss implications of the results for practice, policy, and future research.	Discussion, page 13
**OTHER INFORMATION**			
**Registration and protocol**	24a	Provide registration information for the review, including register name and registration number, or state that the review was not registered.	Methods, page 5
	24b	Indicate where the review protocol can be accessed, or state that a protocol was not prepared.	Methods, page 5
	24c	Describe and explain any amendments to information provided at registration or in the protocol.	Methods, page 5
**Support**	25	Describe sources of financial or non-financial support for the review, and the role of the funders or sponsors in the review.	Page 3
**Competing interests**	26	Declare any competing interests of review authors.	Page 3
**Availability of data, code and other materials**	27	Report which of the following are publicly available and where they can be found: template data collection forms; data extracted from included studies; data used for all analyses; analytic code; any other materials used in the review.	Data extracted from included studies - Supplemental Materials

Topic	No.	Item	Reported?
**TITLE**			
**Title**	1	Identify the report as a systematic review.	Yes
**BACKGROUND**			
**Objectives**	2	Provide an explicit statement of the main objective(s) or question(s) the review addresses.	Yes
**METHODS**			
**Eligibility criteria**	3	Specify the inclusion and exclusion criteria for the review.	Yes
**Information sources**	4	Specify the information sources (e.g. databases, registers) used to identify studies and the date when each was last searched.	Yes
**Risk of bias**	5	Specify the methods used to assess risk of bias in the included studies.	Yes
**Synthesis of results**	6	Specify the methods used to present and synthesize results.	Yes
**RESULTS**			
**Included studies**	7	Give the total number of included studies and participants and summarise relevant characteristics of studies.	Yes
**Synthesis of results**	8	Present results for main outcomes, preferably indicating the number of included studies and participants for each. If meta-analysis was done, report the summary estimate and confidence/credible interval. If comparing groups, indicate the direction of the effect (i.e. which group is favoured).	Yes
**DISCUSSION**			
**Limitations of evidence**	9	Provide a brief summary of the limitations of the evidence included in the review (e.g. study risk of bias, inconsistency and imprecision).	Yes
**Interpretation**	10	Provide a general interpretation of the results and important implications.	Yes
**OTHER**			
**Funding**	11	Specify the primary source of funding for the review.	Not applicable
**Registration**	12	Provide the register name and registration number.	Yes

Supplemental Table 2: Search strategy for each database

Database	#	Search Query	Results
Web of Science Core Collection	1	((((((((ALL=("pregnan*")) OR ALL=("maternal")) OR ALL=("antenatal")) OR ALL=("perinatal")) OR ALL=("childbirth")) OR ALL=("pregnant woman")) OR ALL=("pregnant women")) OR ALL=("expectant mother*")) OR ALL=("pregnant people")	1,135,800
Web of Science Core Collection	2	(((((((ALL=("multi-morbid*")) OR ALL=("co-morbid*")) OR ALL=("multimorbid*")) OR ALL=("comorbid*")) OR ALL=("multiple chronic condition*")) OR ALL=("multiple chronic disease*")) OR ALL=("multiple long term condition*")) OR ALL=("multiple disease*")	367,181
Web of Science Core Collection	3	ALL=("prevalence*")	1,374,916
Web of Science Core Collection	4	#3 AND #2 AND #1 Timespan: 2015-01-01 to 2025-02-10	**1,895**
EMBASE	1	prevalence*	1,545,660
EMBASE	2	pregnan*' OR 'pregnancy'/exp OR 'maternal' OR 'antenatal' OR 'perinatal' OR 'childbirth'/exp OR 'childbirth' OR 'pregnant woman' OR 'pregnant women' OR 'expectant mother'/exp OR 'expectant mother' OR 'pregnant people'	1,695,737
EMBASE	3	multi morbid*' OR 'co morbid*' OR 'multimorbid*' OR 'comorbid*' OR 'multiple chronic condition*' OR 'multiple disease*' OR 'multiple long term condition*' OR 'multiple chronic conditions'/exp	718,466
EMBASE	4	#1 AND #2 AND #3	4,148
EMBASE	5	#1 AND #2 AND #3 AND [01-01-2015]/sd NOT [11-02-2025]/sd	3,136
EMBASE	8	#1 AND #2 AND #3 AND [01-01-2015]/sd NOT [11-02-2025]/sd AND [embase]/lim	**2,936**
MEDLINE	1	exp Prevalence/	375,268
MEDLINE	2	prevalence*.mp.	1,013,114
MEDLINE	3	1 or 2	1,013,114
MEDLINE	4	maternal.mp.	410,513
MEDLINE	5	antenatal.mp.	51,211
MEDLINE	6	perinatal.mp.	101,106
MEDLINE	7	childbirth.mp.	31,170
MEDLINE	8	exp Pregnant Women/	16,896
MEDLINE	9	pregnant women.mp.	131,558

Supplemental Table 2 (continued)

Database	#	Search Query	Results
MEDLINE	10	pregnant woman.mp.	12,564
MEDLINE	11	pregnan*.mp.	1,213,348
MEDLINE	12	expectant mother.mp.	281
MEDLINE	13	exp Pregnancy/	1,064,068
MEDLINE	14	pregnant people.mp.	18,151
MEDLINE	15	4 or 5 or 6 or 7 or 8 or 9 or 10 or 11 or 12 or 13 or 14	1,392,788
MEDLINE	16	multi-morbid*.mp.	1,493
MEDLINE	17	co-morbid*.mp.	36,570
MEDLINE	18	multimorbid*.mp.	12,810
MEDLINE	19	comorbid*.mp.	356,691
MEDLINE	20	exp Comorbidity/	135,065
MEDLINE	21	multiple chronic condition*.mp.	2,590
MEDLINE	22	exp Multiple Chronic Conditions/	834
MEDLINE	23	multiple disease*.mp.	6,248
MEDLINE	24	multiple long-term condition*.mp.	304
MEDLINE	25	16 or 17 or 18 or 19 or 20 or 21 or 22 or 23 or 24	401,390
MEDLINE	26	3 and 15 and 25	2,070
MEDLINE	27	limit 26 to dt=20150101-20250210 [January 1st, 2015 to February 10th, 2025]	**1,158**

Supplemental Table 3: Study characteristics

Author name and year of publication	Country	Region	World Bank income region	Study start date and end date	Mean age in years	Women in age group category ≥35 years	Conditions	Number of conditions	Total number of participants	Number of pregnant women with multimorbidity	Prevalence of multimorbidity (%)
Abdus-salam et al. 2022	Nigeria	Africa	Low-middle income	2020-2020	32	95 (25.0%)	Depression, anxiety	2	380	15	3.95
Akaishi et al. 2023	Japan	Asia	High income	2016-2021	Not reported	Not reported	Suicide attempt, diabetes	2	804617	94	0.01
Alnasheet et al. 2024	Bahrain	Asia	High income	2023-2023	Not reported	119 (23.2%)	Anxiety, chronic comorbidity ("e.g., diabetes and hypertension" but does not specify further)	2	513	85	16.57
Ayers et al. 2024	UK	Europe	High income	2021-2022	32.4	Not reported	Depression, anxiety	2	303	16	5.28
Bante et al. 2021	Ethiopia	Africa	Low income	2019-2019	27.4	96 (14.4%)	Depression and anxiety with: intimate partner violence	3	667	42	6.30
Belsti et al. 2024	Australia	Oceania	High income	2016-2021	Not reported	10913 (22.5%)	Substance use and two or more pre-existing medical conditions (i.e. obesity, anemia, mental health disorders)	3	48502	3064	6.32
Bitew et al. 2019	Ethiopia	Africa	Low income	2014-2014	26.8	Not reported	Depression symptoms, chronic illness	2	1311	180	13.73
Bjorkstedt et al. 2022	Finland	Europe	High income	2009-2015	28.6	Not reported	Psychiatric diagnosis, smoking during pregnancy	2	6189	64	1.03

Author name and year of publication	Country	Region	World Bank income region	Study start date and end date	Mean age in years	Women in age group category ≥35 years	Conditions	Number of conditions	Total number of participants	Number of pregnant women with multimorbidity	Prevalence of multimorbidity (%)
Bjørk et al. 2015	Norway	Europe	High income	1999-2008	29.7	Not reported	Epilepsy with: peripartum depression	2	107516	189	0.18
Bourjeily et al. 2017	United States	North America	High income	2010-2014	29.6	Not reported	Obstructive sleep apnea (OSA) and tobacco use	2	1577632	176	0.01
Brown et al. 2024	Canada	North America	High income	2007-2020	29.2	69022 (3.42%)	Substance use and presence of another chronic condition	2	2014508	51181	2.54
Cena et al. 2021	Italy	Europe	High income	2017-2019	Not reported	284 (30.41%)	Depression, anxiety	2	934	64	6.85
Chen et al. 2017	United States	North America	High income	2003-2009	26	Not reported	Intimate partner violence and current alcohol use	2	1438	8	0.56
Chen et al. 2023	United States	North America	High income	1998-2018	Not reported	1296209 (1.85%)	Hepatitis C virus (HCV) and tobacco use	2	70038267	57263	0.082
Chen et al. 2023	Taiwan	Asia	High income	2015-2019	Not reported	120792 (20.65%)	Depression symptoms and tobacco use	2	584958	1892	0.32
Chhabria et al. 2024	United States	North America	High income	2014-2019	31.86	Not reported	Metabolic Syndrome Conditions (MetS-C) and one of (depression, bipolar, anxiety, psychosis)	2	372895	10091	2.71

Author name and year of publication	Country	Region	World Bank income region	Study start date and end date	Mean age in years	Women in age group category ≥35 years	Conditions	Number of conditions	Total number of participants	Number of pregnant women with multimorbidity	Prevalence of multimorbidity (%)
Coleman-Cowger et al. 2018	United States	North America	High income	2017-2017	27.9	Not reported	Co-use of tobacco cigarettes and cannabis	2	500	45	9
Conner et al. 2015	United States	North America	High income	2004-2008	24.9	Not reported	Marijuana use and tobacco use	2	8138	395	4.85
Dalton et al. 2023	United States	North America	High income	2021	31.8	23177 (30.59%)	Chronic pain with anxiety and/or depression	2	75768	5815	7.67
David et al. 2023	Brazil	South America	Upper-middle income	2018-2019	Not reported	Not reported	Overweight/obesity and anxiety symptoms	2	1279	205	16.03
David et al. 2023	United States	North America	High income	2015–2019	Not reported	536 (15.9%)	Serious psychological distress, cigarette use	2	3373	89	2.64
Dikmen-Yildiz et al. 2017	Turkey	Asia	Upper-middle income	2014-2015	27.6	107 (11.26%)	Depression, anxiety	2	950	104	10.95
Dindo et al. 2017	United States	North America	High income	2000-2004	25.8	Not reported	Major depressive episode, generalised anxiety disorder	2	5404	47	0.87
Edvardsson et al. 2022	Australia	Oceania	High income	2009-2016	30.9	149869 (25.15%)	Severe mental illness (SMI) with: any mental illness (except SMI)	2	595792	1151	0.19

Author name and year of publication	Country	Region	World Bank income region	Study start date and end date	Mean age in years	Women in age group category ≥35 years	Conditions	Number of conditions	Total number of participants	Number of pregnant women with multimorbidity	Prevalence of multimorbidity (%)
Ehrenthal et al. 2024	United States	North America	High income	2011-2019	Not reported	20001 (9.46%)	Prenatal Opioid Use Disorder, tobacco use	2	216684	3815	1.76
Ertmann et al. 2022	Denmark	Europe	High income	2015-2016	Not reported	238 (18.5%)	Record of mental disease, somatic comorbidities	2	1290	34	2.64
Fabre et al. 2021	France	Europe	High income	2015-2019	30.3	796230 (21.7%)	Schizophrenia, smoking addiction	ICD-10 codes	3667461	447	0.01
Ferrara et al. 2022	United States	North America	High income	2020-2021	30.7	10452 (23.8%)	SARS-CoV-2 infection (COVID-19) and smoking during pregnancy	2	43886	60	0.14
Friedman et al. 2016	Peru	South America	Upper-middle income	2012-2014	28.19	603 (18.1%)	Depression, migraine	2	3323	180	5.42
Girchenko et al. 2018	Finland	Europe	High income	2005-2009	31.8	Not reported	Overweight/obese and alcohol use during pregnancy	2	3117	138	4.43
González-Mesa et al. 2020	Spain and Turkey	Europe and Asia	High income and upper-middle income	Not reported	31	Not reported	Depression, anxiety	2	514	138	26.85
Hartwell et al. 2023	United States	North America	High income	2016-2019	Not reported	Not reported	Intimate partner violence (IPV) and depression	2	140817	2285	1.62

Author name and year of publication	Country	Region	World Bank income region	Study start date and end date	Mean age in years	Women in age group category ≥35 years	Conditions	Number of conditions	Total number of participants	Number of pregnant women with multimorbidity	Prevalence of multimorbidity (%)
Hesselman et al. 2020	Sweden	Europe	High income	2007-2014	28.39	45509 (12.06%)	ADHD, early pregnancy smoking	2	377381	1650	0.44
Heun-Johnson et al. 2019	United States	North America	High income	2008-2014	27.9	Not reported	Severe mental illness SMI) (including major depressive disorder (MDD), bipolar disorder (BD), or schizophrenia) with: tobacco use	2	5518766	12521	0.23
Houtchens et al. 2018	United States	North America	High income	2006-2015	29.5	7442 (17.91%)	Multiple sclerosis (MS), anxiety	2	41553	267	0.64
Huang et al. 2023	United States	North America	High income	2016-2018	Not reported	102305 (18.95%)	Housing-insecure, substance use disorder	2	539950	983	0.18
Hulsbosch et al. 2023	The Netherlands	Europe	High income	2013-2014	30.4	Not reported	Comorbid anxiety and depression (CAD) with: smoking in pregnancy	3	1682	27	1.61
Iacobelli et al. 2017	Réunion Island	Africa	Upper-middle income	2001–2015	27.6	8639 (14.1%)	Preeclampsia and smoking during pregnancy	2	61062	125	0.2
Kassee et al. 2023	Canada	North America	High income	2003-2018	Not reported	230703 (20.44%)	Intellectual and developmental disabilities (IDD), psychiatric comorbidity	2	1128776	791	0.07
Kattini et al. 2020	Canada	North America	High income	2012-2017	24.4	110 (5.31%)	Diabetes (gestational and Type II) and smoking	2	2073	135	6.51

Author name and year of publication	Country	Region	World Bank income region	Study start date and end date	Mean age in years	Women in age group category ≥35 years	Conditions	Number of conditions	Total number of participants	Number of pregnant women with multimorbidity	Prevalence of multimorbidity (%)
Kemppinen et al. 2022	Finland	Europe	High income	2011-2015	Not reported	Not reported	Gestational anaemia, alcohol consumption first trimester	2	1273	46	3.61
Kendle et al. 2022	United States	North America	High income	2006-2017	Not reported	7172471 (15.3%)	Insomnia, major mental health disorder	2	46975745	21186	0.05
Khan et al. 2018	Saudi Arabia	Asia	High income	2014-2014	30.11	Not reported	Depression, restless leg syndrome	2	517	5	0.97
Kishore et al. 2019	United States	North America	High income	2003-2011	27	1246493 (3.1%)	Rheumatoid arthritis (RA) and smoking	2	42317648	1439	3.4
Kolstad et al. 2015	Norway	Europe	High income	1999-2008	Not reported	Not reported	Epilepsy, binge eating disorder during pregnancy	2	107214	41	0.04
Laine et al. 2018	Finland	Europe	High income	2009-2015	28.2	Not reported	Gestational diabetes mellitus (GDM) and smoking	2	7750	215	2.77
Lange et al. 2015	Canada	North America	High income	2003-2012	Not reported	1339 (6.8%)	Smoked during pregnancy and alcohol use during pregnancy	2	22962	444	1.90
Larsen et al. 2016	Denmark	Europe	High income	1996-2002	29.2	Not reported	Eating disorder, alcohol intake during pregnancy	2	83731	130	0.16
Leal et al. 2020	Brazil	South America	Upper-middle income	2014-2016	27.6	Not reported	Hypertensive disorder of pregnancy (HDP), depression or other nervous disorders	2	4262	200	4.69
Lee et al. 2022	UK	Europe	High income	2018-2018	Not reported	7576 (20.1%)	Anxiety, Depression, Other mental health conditions	3	37641	175	0.46

Author name and year of publication	Country	Region	World Bank income region	Study start date and end date	Mean age in years	Women in age group category ≥35 years	Conditions	Number of conditions	Total number of participants	Number of pregnant women with multimorbidity	Prevalence of multimorbidity (%)
Lelisho et al. 2022	Ethiopia	Africa	Low income	2020-2020	31.09	Not reported	Generalized anxiety disorder, chronic illness	2	423	37	8.75
Li et al. 2024	China	Asia	Upper-middle income	2022-2022	29	82 (9.2%)	Anxiety, passive smoking during pregnancy	2	887	53	5.98
Litman et al. 2022	United States	North America	High income	2019-2021	29.6	8830 (20.9%)	COVID-19, major mental illness	2	43612	331	0.76
Liu et al. 2024	China	Asia	Upper-middle income	2024-2024	Not reported	192 (18.3%)	Co-occurring insomnia and anxiety	2	1049	289	27.55
López-de-Andrés et al. 2020	Spain	Europe	High income	2009-2015	31.2	732815 (29.5%)	Diabetes mellitus (Type 1, Type 2, and gestational) and tobacco use	2	2481479	122050	4.91
Lopez-de-Andrés et al. 2024	Spain	Europe	High income	2016–2022	32.1	735644 (38.86%)	Diabetes mellitus (Type 1, Type 2, and gestational) and drug abuse	2	1995953	225	0.01
Lupattelli et al. 2015	Norway	Europe	High income	1999-2008	30	Not reported	Binge-eating disorder (BED), gastrointestinal disorders	2	62019	2159	3.48
Ma et al. 2022	China	Asia	Upper-middle income	2008-2008	Not reported	392 (24.8%)	Comorbid depression and anxiety symptoms, plus alcohol use	3	1583	28	1.77
Maack et al. 2019	Sweden	Europe	High income	2009-2016	31.5	Not reported	Depression and overweight/obesity	2	3965	59	1.49
Magtanong et al. 2019	United States	North America	High income	1999-2013	Not reported	1824381 (14.49%)	Fibromyalgia, major depressive disorder	2	12592676	1144	0.01

Author name and year of publication	Country	Region	World Bank income region	Study start date and end date	Mean age in years	Women in age group category ≥35 years	Conditions	Number of conditions	Total number of participants	Number of pregnant women with multimorbidity	Prevalence of multimorbidity (%)
Margolese et al. 2025	United States	North America	High income	2004-2014	Not reported	1341020 (14.7%)	Epilepsy and tobacco smoking during pregnancy	2	9096788	3435	0.04
Mateus et al. 2022	Brazil, Chile, Cyprus, Greece, Israel, Portugal, Spain, Turkey, and the United Kingdom	South America, Europe, Asia	High income, upper-middle income	2020-2020	31.84	Not reported	Comorbid depression and anxiety	2	3326	504	15.15
Männistö et al. 2016	United States	North America	High income	2002-2008	Not reported	33438 (14.97%)	Depression with anxiety and: underweight	3	223394	251	0.11
Meinhofer et al. 2022	United States	North America	High income	2021-2021	28.24	3252933 (15.6%)	Cannabis use disorder (CUD), and other substance use disorders (SUDs), and mood-related disorders	3	20914591	87181	0.42
Metz et al. 2024	United States	North America	High income	2010-2013	Not reported	Not reported	Cannabis exposure during pregnancy, anxiety	2	9257	218	2.35
Mogos et al. 2019	United States	North America	High income	2002-2014	Not reported	8600808 (14.66%)	Hypertensive disorder and depression with: anemia	3	58679098	13090	0.02

Author name and year of publication	Country	Region	World Bank income region	Study start date and end date	Mean age in years	Women in age group category ≥35 years	Conditions	Number of conditions	Total number of participants	Number of pregnant women with multimorbidity	Prevalence of multimorbidity (%)
Mogos et al. 2023	United States	North America	High income	2016-2020	Not reported	3589169 (18.17%)	Takotsubo cardiomyopathy with: anxiety	2	19754535	95	0.00
Nasiri et al. 2021	United States	North America	High income	1999-2015	Not reported	2005355 (14.54%)	Major depressive disorder, obsessive-compulsive disorder	2	13792544	997	0.01
Nath et al. 2018	UK	Europe	High income	2014-2016	Not reported	41 (7.77%)	Depression, generalised anxiety disorder	2	545	39	7.16
Obrochta et al. 2020	US and Canada	North America	High income	2016-2018	33.15	100 (34.7%)	Depression, stress, anxiety	3	288	10	3.47
Orós et al. 2023	Spain	Europe	High income	2012-2018	Not reported	4365 (25.98%)	Overweight/obese and depression	2	16803	159	0.95
Orta et al. 2015	United States	North America	High income	2009-2013	33.3	479 (36.26%)	Migraine, stress (mild to severe)	2	1321	146	11.05
Pampaka et al. 2018	Kuwait	Asia	High income	2012-2015	28.7	233 (12%)	Overweight/obesity and depressive symptoms	2	1912	209	10.93
Potnuru et al. 2024	United States	North America	High income	2016-2020	29	Not reported	Pulmonary hypertension, major mental health disorder	2	18161315	775	0.00
Premji et al. 2020	Pakistan	Asia	Low-middle income	2015-2016	26.8	Not reported	Comorbid anxiety and depression	2	282	64	22.70
Raina et al. 2021	United States	North America	High income	2004-2014	Not reported	Not reported	Any hypertensive disorders of pregnancy, any mental disorders during pregnancy	2	9097355	15042	0.17

Author name and year of publication	Country	Region	World Bank income region	Study start date and end date	Mean age in years	Women in age group category ≥35 years	Conditions	Number of conditions	Total number of participants	Number of pregnant women with multimorbidity	Prevalence of multimorbidity (%)
Ren et al. 2021	Denmark	Europe	High income	1978–2016	Not reported	59175 (6.3%)	Atopic disease (asthma, atopic dermatitis, allergic rhinitis), mental disorders during pregnancy	2	937422	377	0.04
Salahuddin et al. 2020	United States	North America	High income	2011-2014	Not reported	187366 (13.1%)	Human immunodeficiency virus-positive (HIV positive) and drug or alcohol users	2	1434441	12467	0.87
Shen et al. 2021	United States	North America	High income	2009-2014	Not reported	305644 (20.89%)	Opioid use disorder, other dependence or abuse of psychoactive substances	2	1463302	6509	0.44
Shuffrey et al. 2022	United States	North America	High income	Not reported	30.2	Not reported	Prenatal depression, gestational diabetes, overweight/obesity	3	5822	63	1.08
Skoglund et al. 2019	Sweden	Europe	High income	2005-2014	Not reported	Not reported	ADHD and alcohol and substance use disorder	3	384103	940	0.24
Stanhope et al. 2022	United States	North America	High income	2015-2021	Not reported	2178 (15.31%)	Multimorbidity including psychiatric history	2	14225	595	4.18
Uguz et al. 2019	Turkey	Asia	Upper-middle income	Not reported	28.25	Not reported	At least two psychiatric diagnoses	2	1154	78	6.76
Valinder et al. 2023	Canada	North America	High income	2007-2018	29.67	227976 (0.19%)	Physical disability and smoking during pregnancy	2	1220822	706	0.06
Van Heyningen et al. 2017	South Africa	Africa	Upper-middle income	2011-2012	27	Not reported	Diagnosis with any anxiety disorder, current MDE diagnosis	2	376	45	11.97
Vigod et al. 2019	Canada	North America	High income	2002-2015	Not reported	Not reported	Perinatal psychiatric disorder and inflammatory bowel disease (IBD)	2	802,629	371	0.05

Author name and year of publication	Country	Region	World Bank income region	Study start date and end date	Mean age in years	Women in age group category ≥35 years	Conditions	Number of conditions	Total number of participants	Number of pregnant women with multimorbidity	Prevalence of multimorbidity (%)
Welander et al. 2021	Sweden	Europe	High income	2009-2018	Not reported	1184	Migraine, depression, anxiety	3	4831	62	1.28
Wu et al. 2020	United States	North America	High income	2004-2014	Not reported	Not reported	Stroke, smoking	2	4240284	259	0.01
Yang et al. 2016	Peru	South America	Upper-middle income	2012-2014	28.13	525 (17.93%)	PTSD, depression and obesity	3	2899	64	2.21
Zahid et al. 2024	United States	North America	High income	2016-2020	29.11	Not reported	Cardiovascular complications, depression	2	2371661	9078	0.38
Zhang et al. 2021	China	Asia	Upper-middle income	2020-2020	29.49	Not reported	Depression with: physical comorbidities (one or more)	2	769	16	2.08
Zhang et al. 2025	China	Asia	Upper-middle income	2022-2023	Not reported	Not reported	Comorbid anxiety and depression (CAD)	2	3053	477	15.62

Supplemental Table 4: GRADE summary

**Outcome**	**Prevalence**	**Number of studies**	**Certainty**	**Comments**
Prevalence of mental health-related multimorbidity during pregnancy	1.90% (95% CI: 1.73% -2.07%)	92	⨁⨁◯◯ Low	Downgraded for observational studies and evidence of publication bias, however there is a low risk of bias assessment for most studies. Note: the GRADE approach is not well-suited for systematic reviews of prevalence.

Supplemental Table 5: Quality assessment using the Newcastle-Ottawa Scale

	Selection				Comparability		Outcome			
**Author name and year of publication**	**Representativeness of the exposed cohort**	**Selection of the non-exposed cohort**	**Ascertainment of exposure**	**Demonstration that outcome of interest (maternal multimorbidity) was accounted for or not present at start of study**	**Study controls or accounts for age and accounts for any additional factor**		**Assessment of outcome (maternal multimorbidity)**	**Was follow-up long enough for outcomes to occur**	**Adequacy of follow up of cohorts**	**Total ***
Abdus-salam et al. 2022	*	*	*	-	*	*	-	-	-	5
Akaishi et al. 2023	*	*	*	-	-	*	*	*	-	6
Alnasheet et al. 2024	*	*	*	-	*	*	-	-	-	5
Ayers et al. 2024	*	*	*	-	-	-	*	*	-	5
Bante et al. 2021	*	*	*	-	-	-	-	-	*	4
Belsti et al. 2024	*	*	*	-	*	*	*	*	-	7
Bitew et al. 2019	*	*	*	-	-	-	-	*	*	5
Bjorkstedt et al. 2022	*	*	*	-	*	*	*	*	*	8
Bjørk et al. 2015	*	*	-	-	-	*	-	*	*	5
Bourjeily et al. 2017	*	*	*	-	*	*	*	*	-	7

	Selection				Comparability		Outcome			
Brown et al. 2024	*	*	*	-	*	*	*	*	*	8
Cena et al. 2021	*	*	-	-	*	*	-	-	*	5
Chen et al. 2017	*	*	*	-	*	*	*	*	*	7
Chen at al. 2023	*	*	*	-	*	*	*	-	-	6
Chen et al. 2023	-	*	*	-	*	*	*	*	-	6
Chhabria et al. 2024	*	*	*	-	*	*	*	*	*	8
Coleman-Cowger et al. 2018	-	*	*	-	*	*	*	*	*	7
Conner et al. 2015	*	*	*	-	*	*	*	*	-	7
Dalton et al. 2023	*	*	*	-	*	*	*	*	*	8
David et al. 2023	*	*	*	-	*	*	-	-	-	5
David et al. 2023	*	*	*	-	*	*	-	-	-	5
Dikmen-Yildiz et al. 2017	*	*	-	-	-	*	-	*	*	5
Dindo et al. 2017	*	*	*	-	*	*	*	-	-	6
Edvardsson et al. 2022	*	*	*	-	*	*	*	*	*	8

	Selection				Comparability		Outcome			
Ehrenthal et al. 2024	*	*	*	-	*	*	*	*	*	8
Ertmann et al. 2022	*	*	*	-	*	*	*	*	*	8
Fabre et al. 2021	*	*	*	-	*	*	*	*	*	8
Ferrara et al. 2022	*	*	*	-	*	*	*	*	*	8
Friedman et al. 2016	*	*	*	-	*	*	-	-	*	6
Girchenko et al. 2018	*	*	*	-	*	*	*	*	*	8
González-Mesa et al. 2020	-	*	*	-	*	*	-	-	-	4
Hartwell et al. 2023	*	*	-	-	*	*	-	-	*	5
Hesselman et al. 2020	*	*	*	-	*	*	*	*	*	8
Heun-Johnson et al. 2019	*	*	*	-	*	*	*	-	*	7
Houtchens et al. 2018	*	*	*	-	*	*	*	*	-	7
Huang et al. 2023	*	*	*	-	*	*	*	*	-	7
Hulsbosch et al. 2023	*	*	-	-	*	*	-	*	*	6
Iacobelli et al. 2017	*	*	*	-	*	*	*	*	-	7
Kassee et al. 2023	*	*	*	-	*	*	*	*	-	7

	Selection				Comparability		Outcome			
Kattini et al. 2020	*	*	*	-	*	*	*	*	*	8
Kemppinen et al. 2022	*	*	-	-	*	*	-	*	-	5
Kendle et al. 2022	*	*	*	-	*	*	*	-	*	7
Khan et al. 2018	*	*	*	-	*	*	-	-	-	5
Kishore et al. 2019	*	*	*	-	*	*	*	-	*	7
Kolstad et al. 2015	*	*	-	-	-	-	-	*	-	3
Laine et al. 2018	*	*	*	-	*	*	*	*	-	7
Lange et al. 2015	*	*	*	-	*	*	-	-	*	6
Larsen et al. 2016	*	*	*	-	*	*	*	*	-	7
Leal et al. 2020	*	*	*	-	-	-	-	*	*	5
Lee et al. 2022	*	*	*	-	*	*	*	-	*	7
Lelisho et al. 2022	*	*	-	-	-	-	-	-	-	2
Li et al. 2024	-	*	*	-	*	*	-	-	-	4
Litman et al. 2022	*	*	*	-	*	*	*	*	*	8

	Selection				Comparability		Outcome			
Liu et al. 2024	-	*	-	-	*	*	-	-	*	4
López-de-Andrés et al. 2020	*	*	*	-	*	*	*	*	-	7
Lopez-de-Andrés et al. 2024	*	*	*	-	*	*	*	*	-	7
Lupattelli et al. 2015	*	*	-	-	-	-	-	*	*	4
Ma et al. 2022	*	*	*	-	*	*	-	*	*	7
Maack et al. 2019	*	*	-	-	*	*	-	*	-	5
Magtanong et al. 2019	*	*	*	-	*	*	*	*	-	7
Margolese et al. 2025	*	*	*	-	*	*	*	*	*	8
Mateus et al. 2022	-	*	-	-	-	-	-	-	*	2
Männistö et al. 2016	*	*	*	-	*	*	*	*	*	8
Meinhofer et al. 2022	*	*	*	-	-	-	*	*	-	5
Metz et al. 2024	*	*	*	-	-	-	*	*	*	6
Mogos et al. 2019	*	*	*	-	*	*	*	*	*	8
Mogos et al. 2023	*	*	*	-	*	*	*	*	*	8
Nasiri et al. 2021	*	*	*	-	-	*	*	*	-	6
Nath et al. 2018	*	*	*	-	*	*	*	-	*	7

	Selection				Comparability		Outcome			
Obrochta et al. 2020	*	*	*	-	*	*	-	*	*	7
Orós et al. 2023	*	*	*	-	*	*	*	*	*	8
Orta et al. 2015	*	*	*	-	-	*	-	*	-	5
Pampaka et al. 2018	*	*	*	-	*	*	-	*	*	7
Potnuru et al. 2024	*	*	*	-	*	*	*	*	*	8
Premji et al. 2020	-	*	-	-	-	-	-	*	*	3
Raina et al. 2021	*	*	*	-	*	*	*	*	-	7
Ren et al. 2021	*	*	*	-	-	-	*	*	*	6
Salahuddin et al. 2020	*	*	*	-	-	-	*	-	*	5
Shen et al. 2021	*	*	*	-	-	-	*	*	*	6
Shuffrey et al. 2022	*	*	-	-	-	*	-	*	*	5
Skoglund et al. 2019	*	*	*	-	*	*	*	*	*	8
Stanhope et al. 2022	*	*	*	-	-	-	*	*	*	6
Uguz et al. 2019	-	*	*	-	*	*	*	*	*	7

	**Selection**				**Comparability**		**Outcome**			
Valinder et al. 2023	*	*	*	-	*	*	*	*	*	8
Van Heyningen et al. 2017	*	*	*	-	*	*	*	-	-	6
Vigod et al. 2019	*	*	*	-	*	*	*	*	*	8
Welander et al. 2021	*	*	-	-	*	*	-	*	-	5
Wu et al. 2020	*	*	*	-	-	-	*	*	*	6
Yang et al. 2016	*	*	*	-	*	*	-	*	*	7
Zahid et al. 2024	*	*	*	-	*	*	*	*	*	8
Zhang et al. 2021	-	*	-	-	*	*	-	-	*	4
Zhang et al. 2025	*	*	-	*	*	*	-	*	-	6

Supplemental Figure 1: Funnel plot

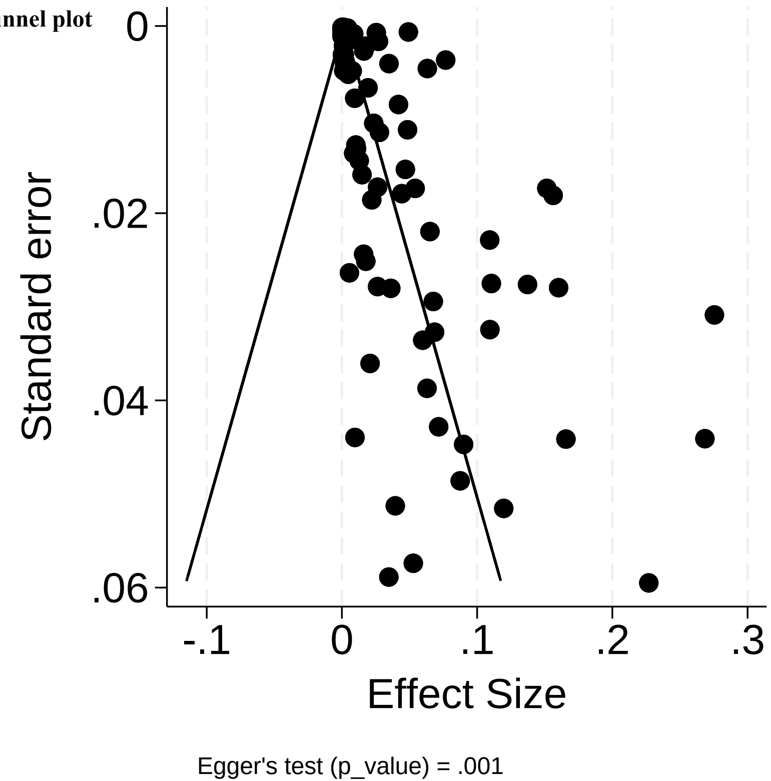

Supplemental Figure 2: Subgroup analysis by study design

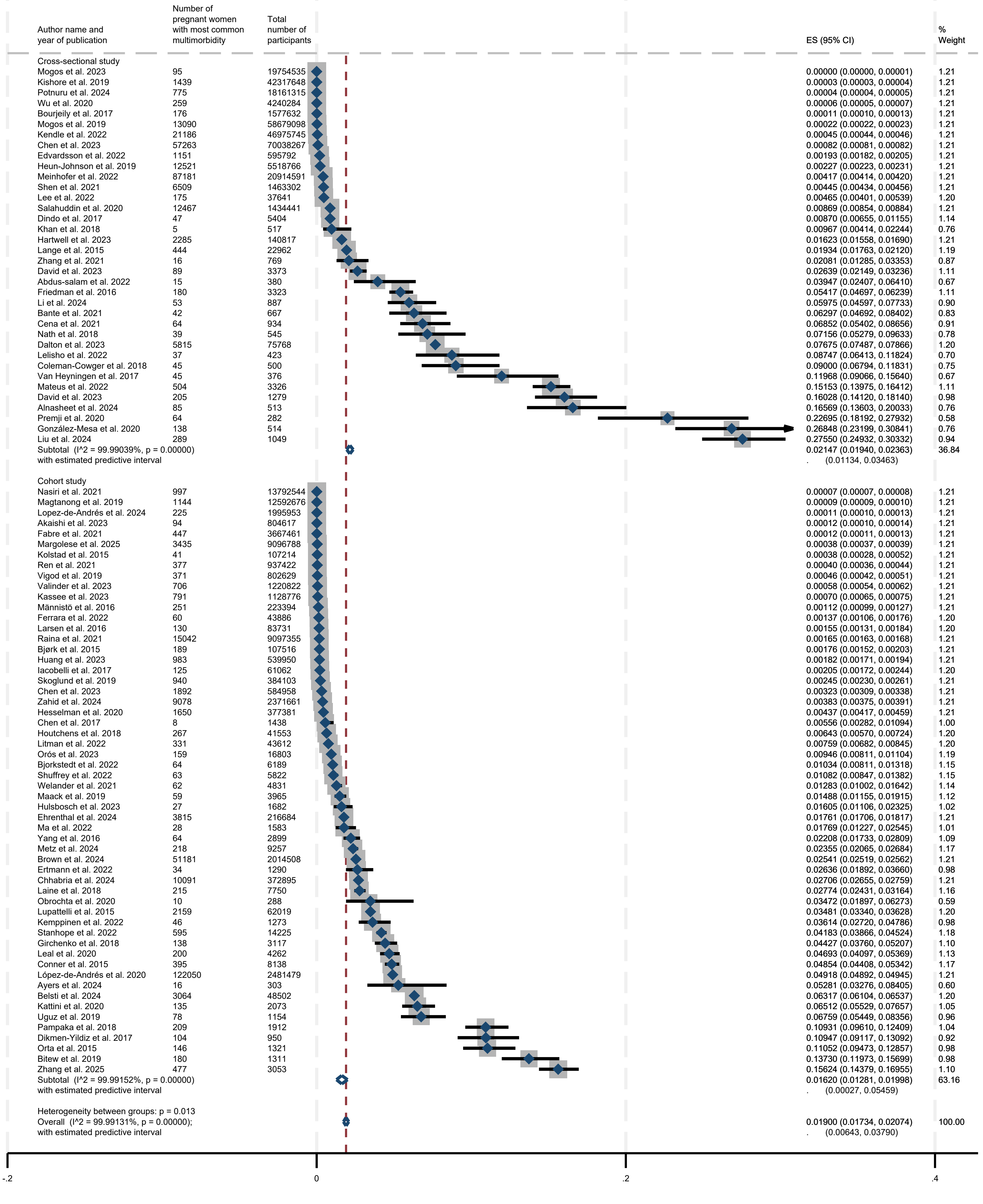

Supplemental Figure 3: Subgroup analysis by income region

Author name and year of publication	Number of pregnant women with most common multimorbidity	Total number of participants	ES (95% CI)	% Weight
High income				
Mogos et al. 2023	95	19754535	0.00000 (0.00000, 0.00001)	1.23
Kishore et al. 2019	1439	42317648	0.00003 (0.00003, 0.00004)	1.23
Potnuru et al. 2024	775	18161315	0.00004 (0.00004, 0.00005)	1.23
Wu et al. 2020	259	4240284	0.00006 (0.00005, 0.00007)	1.23
Nasiri et al. 2021	997	13792544	0.00007 (0.00007, 0.00008)	1.23
Magtanong et al. 2019	1144	12592676	0.00009 (0.00009, 0.00010)	1.23
Bourjeily et al. 2017	176	1577632	0.00011 (0.00010, 0.00013)	1.23
Lopez-de-Andrés et al. 2024	225	1995953	0.00011 (0.00010, 0.00013)	1.23
Akaishi et al. 2023	94	804617	0.00012 (0.00010, 0.00014)	1.23
Fabre et al. 2021	447	3667461	0.00012 (0.00011, 0.00013)	1.23
Mogos et al. 2019	13090	58679098	0.00022 (0.00022, 0.00023)	1.23
Margolese et al. 2025	3435	9096788	0.00038 (0.00037, 0.00039)	1.23
Kolstad et al. 2015	41	107214	0.00038 (0.00028, 0.00052)	1.23
Ren et al. 2021	377	937422	0.00040 (0.00036, 0.00044)	1.23
Kendle et al. 2022	21186	46975745	0.00045 (0.00044, 0.00046)	1.23
Vigod et al. 2019	371	802629	0.00046 (0.00042, 0.00051)	1.23
Valinder et al. 2023	706	1220822	0.00058 (0.00054, 0.00062)	1.23
Kassee et al. 2023	791	1128776	0.00070 (0.00065, 0.00075)	1.23
Chen et al. 2023	57263	70038267	0.00082 (0.00081, 0.00082)	1.23
Männistö et al. 2016	251	223394	0.00112 (0.00099, 0.00127)	1.23
Ferrara et al. 2022	60	43886	0.00137 (0.00106, 0.00176)	1.22
Larsen et al. 2016	130	83731	0.00155 (0.00131, 0.00184)	1.23
Raina et al. 2021	15042	9097355	0.00165 (0.00163, 0.00168)	1.23
Bjørk et al. 2015	189	107516	0.00176 (0.00152, 0.00203)	1.23
Huang et al. 2023	983	539950	0.00182 (0.00171, 0.00194)	1.23
Edvardsson et al. 2022	1151	595792	0.00193 (0.00182, 0.00205)	1.23
Heun-Johnson et al. 2019	12521	5518766	0.00227 (0.00223, 0.00231)	1.23
Skoglund et al. 2019	940	384103	0.00245 (0.00230, 0.00261)	1.23
Chen et al. 2023	1892	584958	0.00323 (0.00309, 0.00338)	1.23
Zahid et al. 2024	9078	2371661	0.00383 (0.00375, 0.00391)	1.23
Meinhofer et al. 2022	87181	20914591	0.00417 (0.00414, 0.00420)	1.23
Hesselman et al. 2020	1650	377381	0.00437 (0.00417, 0.00459)	1.23
Shen et al. 2021	6509	1463302	0.00445 (0.00434, 0.00456)	1.23
Lee et al. 2022	175	37641	0.00465 (0.00401, 0.00539)	1.22
Chen et al. 2017	8	1438	0.00556 (0.00282, 0.01094)	1.02
Houtchens et al. 2018	267	41553	0.00643 (0.00570, 0.00724)	1.22
Litman et al. 2022	331	43612	0.00759 (0.00682, 0.00845)	1.22
Salahuddin et al. 2020	12467	1434441	0.00869 (0.00854, 0.00884)	1.23
Dindo et al. 2017	47	5404	0.00870 (0.00655, 0.01155)	1.17
Orós et al. 2023	159	16803	0.00946 (0.00811, 0.01104)	1.21
Khan et al. 2018	5	517	0.00967 (0.00414, 0.02244)	0.78
Bjorkstedt et al. 2022	64	6189	0.01034 (0.00811, 0.01318)	1.17
Shuffrey et al. 2022	63	5822	0.01082 (0.00847, 0.01382)	1.17
Welander et al. 2021	62	4831	0.01283 (0.01002, 0.01642)	1.16
Maack et al. 2019	59	3965	0.01488 (0.01155, 0.01915)	1.14
Hulsbosch et al. 2023	27	1682	0.01605 (0.01106, 0.02325)	1.04
Hartwell et al. 2023	2285	140817	0.01623 (0.01558, 0.01690)	1.23
Ehrenthal et al. 2024	3815	216684	0.01761 (0.01706, 0.01817)	1.23
Lange et al. 2015	444	22962	0.01934 (0.01763, 0.02120)	1.22
Metz et al. 2024	218	9257	0.02355 (0.02065, 0.02684)	1.19
Brown et al. 2024	51181	2014508	0.02541 (0.02519, 0.02562)	1.23
Ertmann et al. 2022	34	1290	0.02636 (0.01892, 0.03660)	1.00
David et al. 2023	89	3373	0.02639 (0.02149, 0.03236)	1.13
Chhabria et al. 2024	10091	372895	0.02706 (0.02655, 0.02759)	1.23
Laine et al. 2018	215	7750	0.02774 (0.02431, 0.03164)	1.19
Obrochta et al. 2020	10	288	0.03472 (0.01897, 0.06273)	0.60
Lupattelli et al. 2015	2159	62019	0.03481 (0.03340, 0.03628)	1.23
Kemppinen et al. 2022	46	1273	0.03614 (0.02720, 0.04786)	0.99
Stanhope et al. 2022	595	14225	0.04183 (0.03866, 0.04524)	1.21
Girchenko et al. 2018	138	3117	0.04427 (0.03760, 0.05207)	1.12
Conner et al. 2015	395	8138	0.04854 (0.04408, 0.05342)	1.19
López-de-Andrés et al. 2020	122050	2481479	0.04918 (0.04892, 0.04945)	1.23
Ayers et al. 2024	16	303	0.05281 (0.03276, 0.08405)	0.62
Belsti et al. 2024	3064	48502	0.06317 (0.06104, 0.06537)	1.22
Kattini et al. 2020	135	2073	0.06512 (0.05529, 0.07657)	1.07
Cena et al. 2021	64	934	0.06852 (0.05402, 0.08656)	0.93
Nath et al. 2018	39	545	0.07156 (0.05279, 0.09633)	0.79
Dalton et al. 2023	5815	75768	0.07675 (0.07487, 0.07866)	1.23
Coleman-Cowger et al. 2018	45	500	0.09000 (0.06794, 0.11831)	0.77
Pampaka et al. 2018	209	1912	0.10931 (0.09610, 0.12409)	1.06
Orta et al. 2015	146	1321	0.11052 (0.09473, 0.12857)	1.00
Alnasheet et al. 2024	85	513	0.16569 (0.13603, 0.20033)	0.77
Subtotal (I^2 = 99.99316%, p = 0.00000)			0.01057 (0.00921, 0.01202)	83.05
with estimated predictive interval			. (0.00199, 0.02558)	
Upper-middle income				
Iacobelli et al. 2017	125	61062	0.00205 (0.00172, 0.00244)	1.23
Ma et al. 2022	28	1583	0.01769 (0.01227, 0.02545)	1.03
Zhang et al. 2021	16	769	0.02081 (0.01285, 0.03353)	0.88
Yang et al. 2016	64	2899	0.02208 (0.01733, 0.02809)	1.11
Leal et al. 2020	200	4262	0.04693 (0.04097, 0.05369)	1.15
Friedman et al. 2016	180	3323	0.05417 (0.04697, 0.06239)	1.13
Li et al. 2024	53	887	0.05975 (0.04597, 0.07733)	0.92
Uguz et al. 2019	78	1154	0.06759 (0.05449, 0.08356)	0.97
Dikmen-Yildiz et al. 2017	104	950	0.10947 (0.09117, 0.13092)	0.93
Van Heyningen et al. 2017	45	376	0.11968 (0.09066, 0.15640)	0.68
Zhang et al. 2025	477	3053	0.15624 (0.14379, 0.16955)	1.12
David et al. 2023	205	1279	0.16028 (0.14120, 0.18140)	1.00
Liu et al. 2024	289	1049	0.27550 (0.24932, 0.30332)	0.95
Subtotal (I^2 = 99.71914%, p = 0.00000)			0.07052 (0.03053, 0.12524)	13.11
with estimated predictive interval			. (0.00000, 0.37369)	
Low-middle income				
Abdus-salam et al. 2022	15	380	0.03947 (0.02407, 0.06410)	0.68
Premji et al. 2020	64	282	0.22695 (0.18192, 0.27932)	0.59
Subtotal (I^2 = .%, p = .)			0.10293 (0.08078, 0.12738)	1.28
Inestimable predictive distribution with <3 studies			. (- , -)	
Low income				
Bante et al. 2021	42	667	0.06297 (0.04692, 0.08402)	0.85
Lelisho et al. 2022	37	423	0.08747 (0.06413, 0.11824)	0.72
Bitew et al. 2019	180	1311	0.13730 (0.11973, 0.15699)	1.00
Subtotal (I^2 = .%, p = .)			0.09435 (0.05207, 0.14739)	2.56
Inestimable predictive distribution with <3 studies			. (- , -)	
Heterogeneity between groups: p = 0.000				
Overall (I^2 = 99.99149%, p = 0.00000);			0.01737 (0.01576, 0.01904)	100.00
with estimated predictive interval			. (0.00549, 0.03558)	

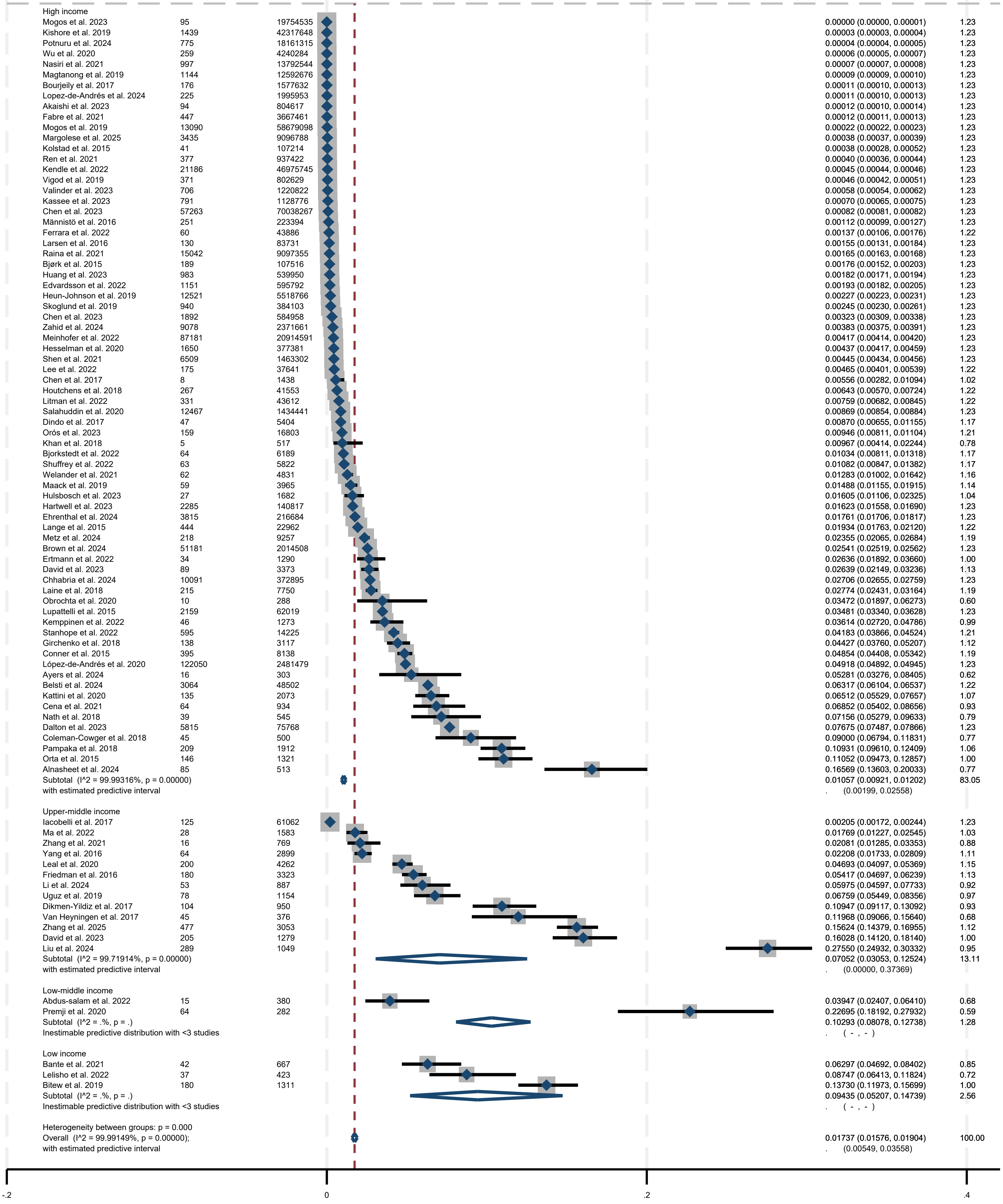

Supplemental Figure 4: Subgroup analysis by number of conditions included in multimorbidity definition

Author name and year of publication	Number of pregnant women with most common multimorbidity	Total number of participants	ES (95% CI)	% Weight
2				
Mogos et al. 2023	95	19754535	0.00000 (0.00000, 0.00001)	1.21
Kishore et al. 2019	1439	42317648	0.00003 (0.00003, 0.00004)	1.21
Potnuru et al. 2024	775	18161315	0.00004 (0.00004, 0.00005)	1.21
Wu et al. 2020	259	4240284	0.00006 (0.00005, 0.00007)	1.21
Nasiri et al. 2021	997	13792544	0.00007 (0.00007, 0.00008)	1.21
Magtanong et al. 2019	1144	12592676	0.00009 (0.00009, 0.00010)	1.21
Bourjeily et al. 2017	176	1577632	0.00011 (0.00010, 0.00013)	1.21
Lopez-de-Andrés et al. 2024	225	1995953	0.00011 (0.00010, 0.00013)	1.21
Akaishi et al. 2023	94	804617	0.00012 (0.00010, 0.00014)	1.21
Fabre et al. 2021	447	3667461	0.00012 (0.00011, 0.00013)	1.21
Margolese et al. 2025	3435	9096788	0.00038 (0.00037, 0.00039)	1.21
Kolstad et al. 2015	41	107214	0.00038 (0.00028, 0.00052)	1.21
Ren et al. 2021	377	937422	0.00040 (0.00036, 0.00044)	1.21
Kendle et al. 2022	21186	46975745	0.00045 (0.00044, 0.00046)	1.21
Vigod et al. 2019	371	802629	0.00046 (0.00042, 0.00051)	1.21
Valinder et al. 2023	706	1220822	0.00058 (0.00054, 0.00062)	1.21
Kassee et al. 2023	791	1128776	0.00070 (0.00065, 0.00075)	1.21
Chen et al. 2023	57263	70038267	0.00082 (0.00081, 0.00082)	1.21
Ferrara et al. 2022	60	43886	0.00137 (0.00106, 0.00176)	1.20
Larsen et al. 2016	130	83731	0.00155 (0.00131, 0.00184)	1.20
Raina et al. 2021	15042	9097355	0.00165 (0.00163, 0.00168)	1.21
Bjørk et al. 2015	189	107516	0.00176 (0.00152, 0.00203)	1.21
Huang et al. 2023	983	539950	0.00182 (0.00171, 0.00194)	1.21
Edvardsson et al. 2022	1151	595792	0.00193 (0.00182, 0.00205)	1.21
Iacobelli et al. 2017	125	61062	0.00205 (0.00172, 0.00244)	1.20
Heun-Johnson et al. 2019	12521	5518766	0.00227 (0.00223, 0.00231)	1.21
Chen et al. 2023	1892	584958	0.00323 (0.00309, 0.00338)	1.21
Zahid et al. 2024	9078	2371661	0.00383 (0.00375, 0.00391)	1.21
Hesselman et al. 2020	1650	377381	0.00437 (0.00417, 0.00459)	1.21
Shen et al. 2021	6509	1463302	0.00445 (0.00434, 0.00456)	1.21
Chen et al. 2017	8	1438	0.00556 (0.00282, 0.01094)	1.00
Houtchens et al. 2018	267	41553	0.00643 (0.00570, 0.00724)	1.20
Litman et al. 2022	331	43612	0.00759 (0.00682, 0.00845)	1.20
Salahuddin et al. 2020	12467	1434441	0.00869 (0.00854, 0.00884)	1.21
Dindo et al. 2017	47	5404	0.00870 (0.00655, 0.01155)	1.14
Orós et al. 2023	159	16803	0.00946 (0.00811, 0.01104)	1.19
Khan et al. 2018	5	517	0.00967 (0.00414, 0.02244)	0.76
Bjorkstedt et al. 2022	64	6189	0.01034 (0.00811, 0.01318)	1.15
Maack et al. 2019	59	3965	0.01488 (0.01155, 0.01915)	1.12
Hartwell et al. 2023	2285	140817	0.01623 (0.01558, 0.01690)	1.21
Ehrenthal et al. 2024	3815	216684	0.01761 (0.01706, 0.01817)	1.21
Lange et al. 2015	444	22962	0.01934 (0.01763, 0.02120)	1.19
Zhang et al. 2021	16	769	0.02081 (0.01285, 0.03353)	0.87
Metz et al. 2024	218	9257	0.02355 (0.02065, 0.02684)	1.17
Brown et al. 2024	51181	2014508	0.02541 (0.02519, 0.02562)	1.21
Ertmann et al. 2022	34	1290	0.02636 (0.01892, 0.03660)	0.98
David et al. 2023	89	3373	0.02639 (0.02149, 0.03236)	1.11
Chhabria et al. 2024	10091	372895	0.02706 (0.02655, 0.02759)	1.21
Laine et al. 2018	215	7750	0.02774 (0.02431, 0.03164)	1.16
Lupattelli et al. 2015	2159	62019	0.03481 (0.03340, 0.03628)	1.20
Kemppinen et al. 2022	46	1273	0.03614 (0.02720, 0.04786)	0.98
Abdus-salam et al. 2022	15	380	0.03947 (0.02407, 0.06410)	0.67
Stanhope et al. 2022	595	14225	0.04183 (0.03866, 0.04524)	1.18
Girchenko et al. 2018	138	3117	0.04427 (0.03760, 0.05207)	1.10
Leal et al. 2020	200	4262	0.04693 (0.04097, 0.05369)	1.13
Conner et al. 2015	395	8138	0.04854 (0.04408, 0.05342)	1.17
López-de-Andrés et al. 2020	122050	2481479	0.04918 (0.04892, 0.04945)	1.21
Ayers et al. 2024	16	303	0.05281 (0.03276, 0.08405)	0.60
Friedman et al. 2016	180	3323	0.05417 (0.04697, 0.06239)	1.11
Li et al. 2024	53	887	0.05975 (0.04597, 0.07733)	0.90
Kattini et al. 2020	135	2073	0.06512 (0.05529, 0.07657)	1.05
Uguz et al. 2019	78	1154	0.06759 (0.05449, 0.08356)	0.96
Cena et al. 2021	64	934	0.06852 (0.05402, 0.08656)	0.91
Nath et al. 2018	39	545	0.07156 (0.05279, 0.09633)	0.78
Dalton et al. 2023	5815	75768	0.07675 (0.07487, 0.07866)	1.20
Lelisho et al. 2022	37	423	0.08747 (0.06413, 0.11824)	0.70
Coleman-Cowger et al. 2018	45	500	0.09000 (0.06794, 0.11831)	0.75
Pampaka et al. 2018	209	1912	0.10931 (0.09610, 0.12409)	1.04
Dikmen-Yildiz et al. 2017	104	950	0.10947 (0.09117, 0.13092)	0.92
Orta et al. 2015	146	1321	0.11052 (0.09473, 0.12857)	0.98
Van Heyningen et al. 2017	45	376	0.11968 (0.09066, 0.15640)	0.67
Bitew et al. 2019	180	1311	0.13730 (0.11973, 0.15699)	0.98
Mateus et al. 2022	504	3326	0.15153 (0.13975, 0.16412)	1.11
Zhang et al. 2025	477	3053	0.15624 (0.14379, 0.16955)	1.10
David et al. 2023	205	1279	0.16028 (0.14120, 0.18140)	0.98
Alnasheet et al. 2024	85	513	0.16569 (0.13603, 0.20033)	0.76
Premji et al. 2020	64	282	0.22695 (0.18192, 0.27932)	0.58
González-Mesa et al. 2020	138	514	0.26848 (0.23199, 0.30841)	0.76
Liu et al. 2024	289	1049	0.27550 (0.24932, 0.30332)	0.94
Subtotal (I^2 = 99.99109%, p = 0.00000)			0.02052 (0.01857, 0.02256)	85.93
with estimated predictive interval			. (0.00681, 0.04124)	
3				
Mogos et al. 2019	13090	58679098	0.00022 (0.00022, 0.00023)	1.21
Männistö et al. 2016	251	223394	0.00112 (0.00099, 0.00127)	1.21
Skoglund et al. 2019	940	384103	0.00245 (0.00230, 0.00261)	1.21
Meinhofer et al. 2022	87181	20914591	0.00417 (0.00414, 0.00420)	1.21
Lee et al. 2022	175	37641	0.00465 (0.00401, 0.00539)	1.20
Shuffrey et al. 2022	63	5822	0.01082 (0.00847, 0.01382)	1.15
Welander et al. 2021	62	4831	0.01283 (0.01002, 0.01642)	1.14
Hulsbosch et al. 2023	27	1682	0.01605 (0.01106, 0.02325)	1.02
Ma et al. 2022	28	1583	0.01769 (0.01227, 0.02545)	1.01
Yang et al. 2016	64	2899	0.02208 (0.01733, 0.02809)	1.09
Obrochta et al. 2020	10	288	0.03472 (0.01897, 0.06273)	0.59
Bante et al. 2021	42	667	0.06297 (0.04692, 0.08402)	0.83
Belsti et al. 2024	3064	48502	0.06317 (0.06104, 0.06537)	1.20
Subtotal (I^2 = 99.99268%, p = 0.00000)			0.01290 (0.00868, 0.01791)	14.07
with estimated predictive interval			. (0.00084, 0.03804)	
Heterogeneity between groups: p = 0.007				
Overall (I^2 = 99.99131%, p = 0.00000);			0.01900 (0.01734, 0.02074)	100.00
with estimated predictive interval			. (0.00643, 0.03790)	

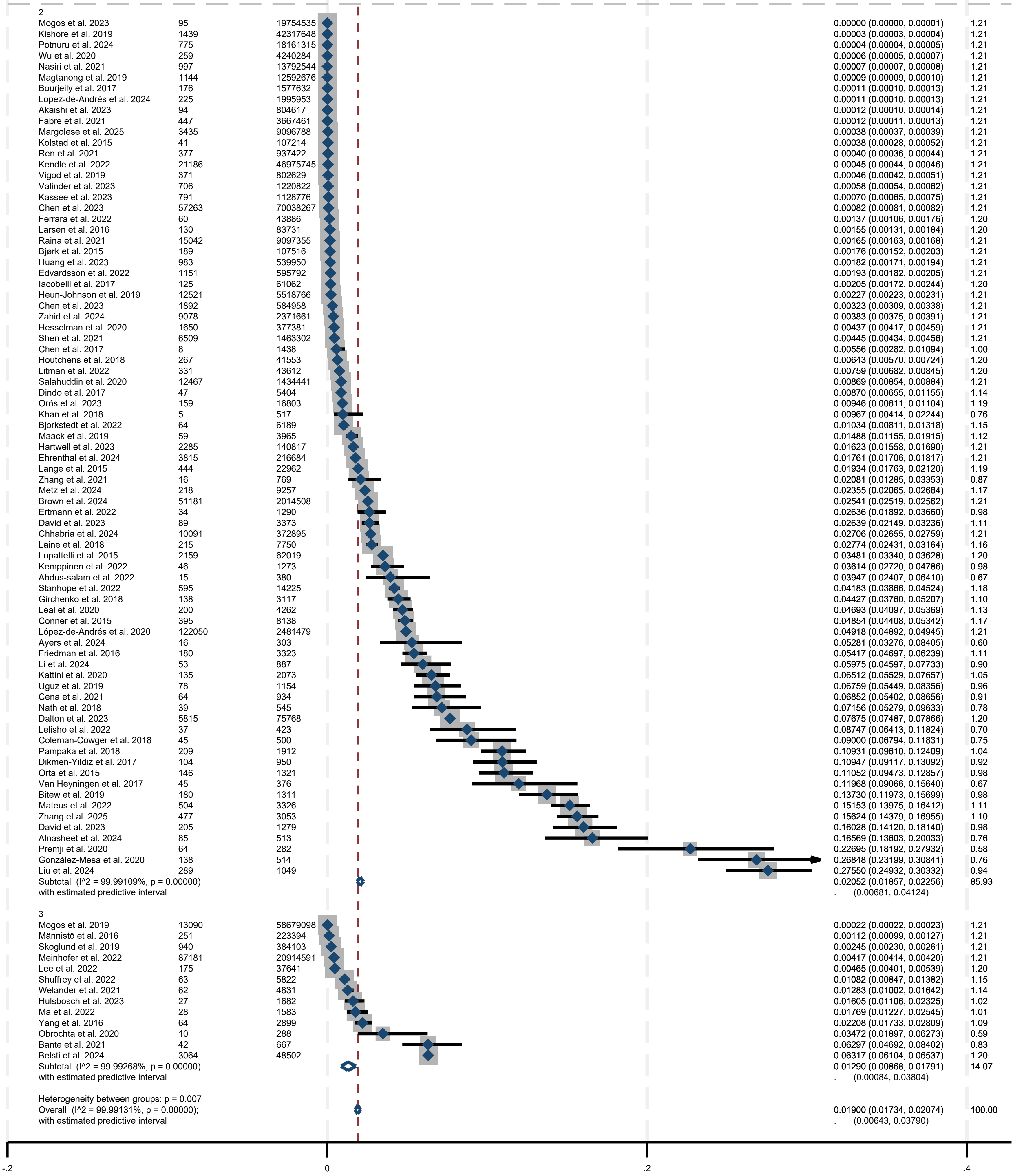

Supplemental Figure 5: Study analysis by presence of depression in reported mental health-related multimorbidity during pregnancy

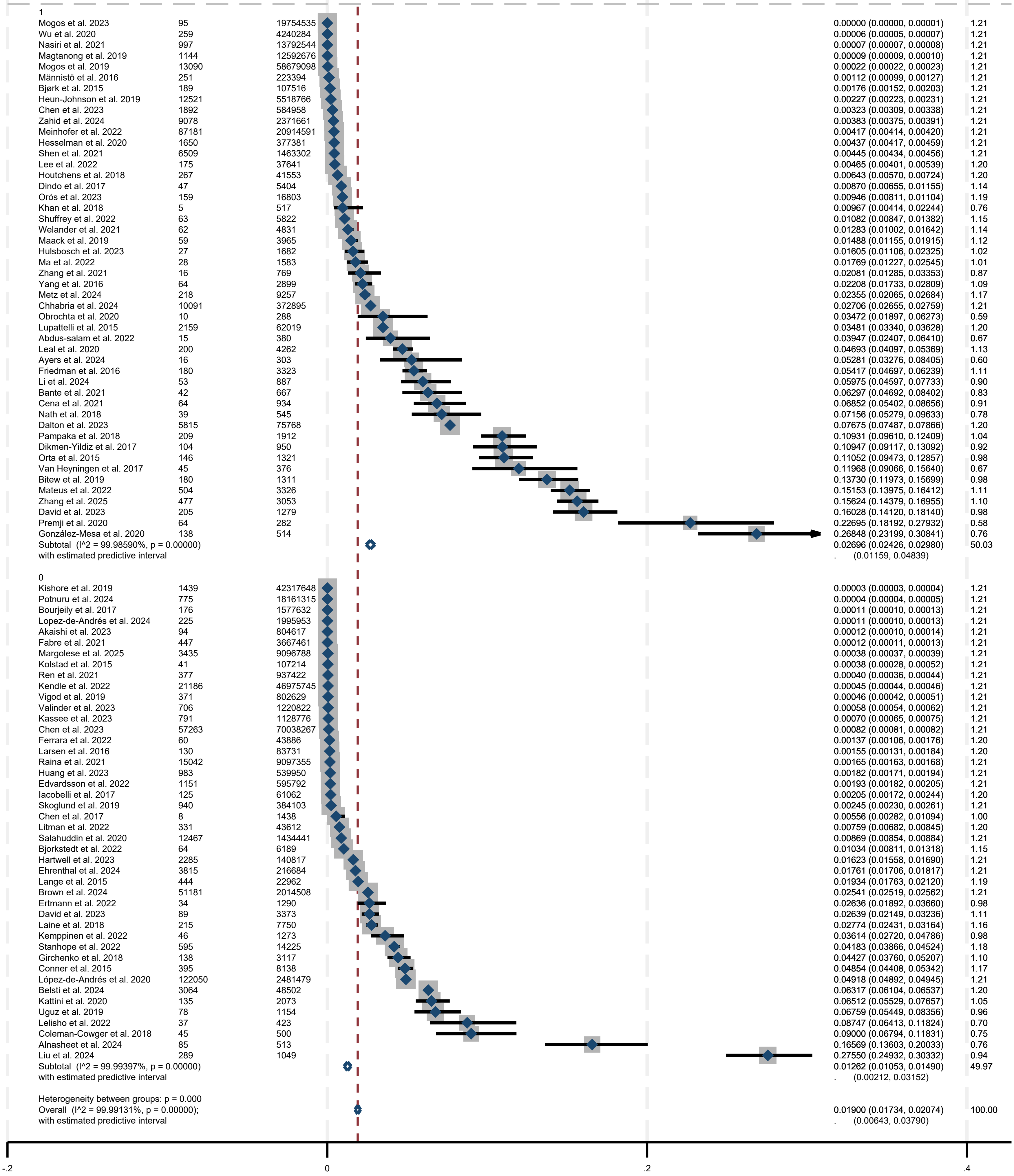

Author name and year of publication	Number of pregnant women with most common multimorbidity	Total number of participants	ES (95% CI)	% Weight
1				
Mogos et al. 2023	95	19754535	0.00000 (0.00000, 0.00001)	1.21
Wu et al. 2020	259	4240284	0.00006 (0.00005, 0.00007)	1.21
Nasiri et al. 2021	997	13792544	0.00007 (0.00007, 0.00008)	1.21
Magtanong et al. 2019	1144	12592676	0.00009 (0.00009, 0.00010)	1.21
Mogos et al. 2019	13090	58679098	0.00022 (0.00022, 0.00023)	1.21
Männistö et al. 2016	251	223394	0.00112 (0.00099, 0.00127)	1.21
Bjørk et al. 2015	189	107516	0.00176 (0.00152, 0.00203)	1.21
Heun-Johnson et al. 2019	12521	5518766	0.00227 (0.00223, 0.00231)	1.21
Chen et al. 2023	1892	584958	0.00323 (0.00309, 0.00338)	1.21
Zahid et al. 2024	9078	2371661	0.00383 (0.00375, 0.00391)	1.21
Meinhofer et al. 2022	87181	20914591	0.00417 (0.00414, 0.00420)	1.21
Hesselman et al. 2020	1650	377381	0.00437 (0.00417, 0.00459)	1.21
Shen et al. 2021	6509	1463302	0.00445 (0.00434, 0.00456)	1.21
Lee et al. 2022	175	37641	0.00465 (0.00401, 0.00539)	1.20
Houtchens et al. 2018	267	41553	0.00643 (0.00570, 0.00724)	1.20
Dindo et al. 2017	47	5404	0.00870 (0.00655, 0.01155)	1.14
Orós et al. 2023	159	16803	0.00946 (0.00811, 0.01104)	1.19
Khan et al. 2018	5	517	0.00967 (0.00414, 0.02244)	0.76
Shuffrey et al. 2022	63	5822	0.01082 (0.00847, 0.01382)	1.15
Welander et al. 2021	62	4831	0.01283 (0.01002, 0.01642)	1.14
Maack et al. 2019	59	3965	0.01488 (0.01155, 0.01915)	1.12
Hulsbosch et al. 2023	27	1682	0.01605 (0.01106, 0.02325)	1.02
Ma et al. 2022	28	1583	0.01769 (0.01227, 0.02545)	1.01
Zhang et al. 2021	16	769	0.02081 (0.01285, 0.03353)	0.87
Yang et al. 2016	64	2899	0.02208 (0.01733, 0.02809)	1.09
Metz et al. 2024	218	9257	0.02355 (0.02065, 0.02684)	1.17
Chhabria et al. 2024	10091	372895	0.02706 (0.02655, 0.02759)	1.21
Obrochta et al. 2020	10	288	0.03472 (0.01897, 0.06273)	0.59
Lupattelli et al. 2015	2159	62019	0.03481 (0.03340, 0.03628)	1.20
Abdus-salam et al. 2022	15	380	0.03947 (0.02407, 0.06410)	0.67
Leal et al. 2020	200	4262	0.04693 (0.04097, 0.05369)	1.13
Ayers et al. 2024	16	303	0.05281 (0.03276, 0.08405)	0.60
Friedman et al. 2016	180	3323	0.05417 (0.04697, 0.06239)	1.11
Li et al. 2024	53	887	0.05975 (0.04597, 0.07733)	0.90
Bante et al. 2021	42	667	0.06297 (0.04692, 0.08402)	0.83
Cena et al. 2021	64	934	0.06852 (0.05402, 0.08656)	0.91
Nath et al. 2018	39	545	0.07156 (0.05279, 0.09633)	0.78
Dalton et al. 2023	5815	75768	0.07675 (0.07487, 0.07866)	1.20
Pampaka et al. 2018	209	1912	0.10931 (0.09610, 0.12409)	1.04
Dikmen-Yildiz et al. 2017	104	950	0.10947 (0.09117, 0.13092)	0.92
Orta et al. 2015	146	1321	0.11052 (0.09473, 0.12857)	0.98
Van Heyningen et al. 2017	45	376	0.11968 (0.09066, 0.15640)	0.67
Bitew et al. 2019	180	1311	0.13730 (0.11973, 0.15699)	0.98
Mateus et al. 2022	504	3326	0.15153 (0.13975, 0.16412)	1.11
Zhang et al. 2025	477	3053	0.15624 (0.14379, 0.16955)	1.10
David et al. 2023	205	1279	0.16028 (0.14120, 0.18140)	0.98
Premji et al. 2020	64	282	0.22695 (0.18192, 0.27932)	0.58
González-Mesa et al. 2020	138	514	0.26848 (0.23199, 0.30841)	0.76
Subtotal (I^2 = 99.98590%, p = 0.00000)			0.02696 (0.02426, 0.02980)	50.03
with estimated predictive interval			. (0.01159, 0.04839)	
0				
Kishore et al. 2019	1439	42317648	0.00003 (0.00003, 0.00004)	1.21
Potnuru et al. 2024	775	18161315	0.00004 (0.00004, 0.00005)	1.21
Bourjeily et al. 2017	176	1577632	0.00011 (0.00010, 0.00013)	1.21
Lopez-de-Andrés et al. 2024	225	1995953	0.00011 (0.00010, 0.00013)	1.21
Akaishi et al. 2023	94	804617	0.00012 (0.00010, 0.00014)	1.21
Fabre et al. 2021	447	3667461	0.00012 (0.00011, 0.00013)	1.21
Margolese et al. 2025	3435	9096788	0.00038 (0.00037, 0.00039)	1.21
Kolstad et al. 2015	41	107214	0.00038 (0.00028, 0.00052)	1.21
Ren et al. 2021	377	937422	0.00040 (0.00036, 0.00044)	1.21
Kendle et al. 2022	21186	46975745	0.00045 (0.00044, 0.00046)	1.21
Vigod et al. 2019	371	802629	0.00046 (0.00042, 0.00051)	1.21
Valinder et al. 2023	706	1220822	0.00058 (0.00054, 0.00062)	1.21
Kassee et al. 2023	791	1128776	0.00070 (0.00065, 0.00075)	1.21
Chen et al. 2023	57263	70038267	0.00082 (0.00081, 0.00082)	1.21
Ferrara et al. 2022	60	43886	0.00137 (0.00106, 0.00176)	1.20
Larsen et al. 2016	130	83731	0.00155 (0.00131, 0.00184)	1.20
Raina et al. 2021	15042	9097355	0.00165 (0.00163, 0.00168)	1.21
Huang et al. 2023	983	539950	0.00182 (0.00171, 0.00194)	1.21
Edvardsson et al. 2022	1151	595792	0.00193 (0.00182, 0.00205)	1.21
Iacobelli et al. 2017	125	61062	0.00205 (0.00172, 0.00244)	1.20
Skoglund et al. 2019	940	384103	0.00245 (0.00230, 0.00261)	1.21
Chen et al. 2017	8	1438	0.00556 (0.00282, 0.01094)	1.00
Litman et al. 2022	331	43612	0.00759 (0.00682, 0.00845)	1.20
Salahuddin et al. 2020	12467	1434441	0.00869 (0.00854, 0.00884)	1.21
Bjorkstedt et al. 2022	64	6189	0.01034 (0.00811, 0.01318)	1.15
Hartwell et al. 2023	2285	140817	0.01623 (0.01558, 0.01690)	1.21
Ehrenthal et al. 2024	3815	216684	0.01761 (0.01706, 0.01817)	1.21
Lange et al. 2015	444	22962	0.01934 (0.01763, 0.02120)	1.19
Brown et al. 2024	51181	2014508	0.02541 (0.02519, 0.02562)	1.21
Ertmann et al. 2022	34	1290	0.02636 (0.01892, 0.03660)	0.98
David et al. 2023	89	3373	0.02639 (0.02149, 0.03236)	1.11
Laine et al. 2018	215	7750	0.02774 (0.02431, 0.03164)	1.16
Kemppinen et al. 2022	46	1273	0.03614 (0.02720, 0.04786)	0.98
Stanhope et al. 2022	595	14225	0.04183 (0.03866, 0.04524)	1.18
Girchenko et al. 2018	138	3117	0.04427 (0.03760, 0.05207)	1.10
Conner et al. 2015	395	8138	0.04854 (0.04408, 0.05342)	1.17
López-de-Andrés et al. 2020	122050	2481479	0.04918 (0.04892, 0.04945)	1.21
Belsti et al. 2024	3064	48502	0.06317 (0.06104, 0.06537)	1.20
Kattini et al. 2020	135	2073	0.06512 (0.05529, 0.07657)	1.05
Uguz et al. 2019	78	1154	0.06759 (0.05449, 0.08356)	0.96
Lelisho et al. 2022	37	423	0.08747 (0.06413, 0.11824)	0.70
Coleman-Cowger et al. 2018	45	500	0.09000 (0.06794, 0.11831)	0.75
Alnasheet et al. 2024	85	513	0.16569 (0.13603, 0.20033)	0.76
Liu et al. 2024	289	1049	0.27550 (0.24932, 0.30332)	0.94
Subtotal (I^2 = 99.99397%, p = 0.00000)			0.01262 (0.01053, 0.01490)	49.97
with estimated predictive interval			. (0.00212, 0.03152)	
Heterogeneity between groups: p = 0.000				
Overall (I^2 = 99.99131%, p = 0.00000);			0.01900 (0.01734, 0.02074)	100.00
with estimated predictive interval			. (0.00643, 0.03790)	

Supplemental Figure 6: Subgroup analysis by mental health-only multimorbidity

Author name and year of publication	Number of pregnant women with most common multimorbidity	Total number of participants	ES (95% CI)	% Weight
**Mental health related and physical**				
Mogos et al. 2023	95	19754535	0.00000 (0.00000, 0.00001)	1.21
Kishore et al. 2019	1439	42317648	0.00003 (0.00003, 0.00004)	1.21
Potnuru et al. 2024	775	18161315	0.00004 (0.00004, 0.00005)	1.21
Wu et al. 2020	259	4240284	0.00006 (0.00005, 0.00007)	1.21
Magtanong et al. 2019	1144	12592676	0.00009 (0.00009, 0.00010)	1.21
Lopez-de-Andrés et al. 2024	225	1995953	0.00011 (0.00010, 0.00013)	1.21
Akaishi et al. 2023	94	804617	0.00012 (0.00010, 0.00014)	1.21
Mogos et al. 2019	13090	58679098	0.00022 (0.00022, 0.00023)	1.21
Margolese et al. 2025	3435	9096788	0.00038 (0.00037, 0.00039)	1.21
Kolstad et al. 2015	41	107214	0.00038 (0.00028, 0.00052)	1.21
Ren et al. 2021	377	937422	0.00040 (0.00036, 0.00044)	1.21
Vigod et al. 2019	371	802629	0.00046 (0.00042, 0.00051)	1.21
Valinder et al. 2023	706	1220822	0.00058 (0.00054, 0.00062)	1.21
Chen et al. 2023	57263	70038267	0.00082 (0.00081, 0.00082)	1.21
Männistö et al. 2016	251	223394	0.00112 (0.00099, 0.00127)	1.21
Ferrara et al. 2022	60	43886	0.00137 (0.00106, 0.00176)	1.20
Raina et al. 2021	15042	9097355	0.00165 (0.00163, 0.00168)	1.21
Bjørk et al. 2015	189	107516	0.00176 (0.00152, 0.00203)	1.21
Huang et al. 2023	983	539950	0.00182 (0.00171, 0.00194)	1.21
Iacobelli et al. 2017	125	61062	0.00205 (0.00172, 0.00244)	1.20
Zahid et al. 2024	9078	2371661	0.00383 (0.00375, 0.00391)	1.21
Chen et al. 2017	8	1438	0.00556 (0.00282, 0.01094)	1.00
Houtchens et al. 2018	267	41553	0.00643 (0.00570, 0.00724)	1.20
Litman et al. 2022	331	43612	0.00759 (0.00682, 0.00845)	1.20
Salahuddin et al. 2020	12467	1434441	0.00869 (0.00854, 0.00884)	1.21
Orós et al. 2023	159	16803	0.00946 (0.00811, 0.01104)	1.19
Shuffrey et al. 2022	63	5822	0.01082 (0.00847, 0.01382)	1.15
Welander et al. 2021	62	4831	0.01283 (0.01002, 0.01642)	1.14
Maack et al. 2019	59	3965	0.01488 (0.01155, 0.01915)	1.12
Hartwell et al. 2023	2285	140817	0.01623 (0.01558, 0.01690)	1.21
Zhang et al. 2021	16	769	0.02081 (0.01285, 0.03353)	0.87
Yang et al. 2016	64	2899	0.02208 (0.01733, 0.02809)	1.09
Brown et al. 2024	51181	2014508	0.02541 (0.02519, 0.02562)	1.21
Ertmann et al. 2022	34	1290	0.02636 (0.01892, 0.03660)	0.98
Chhabria et al. 2024	10091	372895	0.02706 (0.02655, 0.02759)	1.21
Laine et al. 2018	215	7750	0.02774 (0.02431, 0.03164)	1.16
Lupattelli et al. 2015	2159	62019	0.03481 (0.03340, 0.03628)	1.20
Kemppinen et al. 2022	46	1273	0.03614 (0.02720, 0.04786)	0.98
Stanhope et al. 2022	595	14225	0.04183 (0.03866, 0.04524)	1.18
Girchenko et al. 2018	138	3117	0.04427 (0.03760, 0.05207)	1.10
Leal et al. 2020	200	4262	0.04693 (0.04097, 0.05369)	1.13
López-de-Andrés et al. 2020	122050	2481479	0.04918 (0.04892, 0.04945)	1.21
Friedman et al. 2016	180	3323	0.05417 (0.04697, 0.06239)	1.11
Bante et al. 2021	42	667	0.06297 (0.04692, 0.08402)	0.83
Belsti et al. 2024	3064	48502	0.06317 (0.06104, 0.06537)	1.20
Kattini et al. 2020	135	2073	0.06512 (0.05529, 0.07657)	1.05
Uguz et al. 2019	78	1154	0.06759 (0.05449, 0.08356)	0.96
Dalton et al. 2023	5815	75768	0.07675 (0.07487, 0.07866)	1.20
Lelisho et al. 2022	37	423	0.08747 (0.06413, 0.11824)	0.70
Pampaka et al. 2018	209	1912	0.10931 (0.09610, 0.12409)	1.04
Orta et al. 2015	146	1321	0.11052 (0.09473, 0.12857)	0.98
Bitew et al. 2019	180	1311	0.13730 (0.11973, 0.15699)	0.98
David et al. 2023	205	1279	0.16028 (0.14120, 0.18140)	0.98
Alnasheet et al. 2024	85	513	0.16569 (0.13603, 0.20033)	0.76
Subtotal (I^2 = 99.99361%, p = 0.00000)			0.01618 (0.01409, 0.01840)	60.88
with estimated predictive interval			. (0.00413, 0.03581)	
**Mental health related only**				
Nasiri et al. 2021	997	13792544	0.00007 (0.00007, 0.00008)	1.21
Bourjeily et al. 2017	176	1577632	0.00011 (0.00010, 0.00013)	1.21
Fabre et al. 2021	447	3667461	0.00012 (0.00011, 0.00013)	1.21
Kendle et al. 2022	21186	46975745	0.00045 (0.00044, 0.00046)	1.21
Kassee et al. 2023	791	1128776	0.00070 (0.00065, 0.00075)	1.21
Larsen et al. 2016	130	83731	0.00155 (0.00131, 0.00184)	1.20
Edvardsson et al. 2022	1151	595792	0.00193 (0.00182, 0.00205)	1.21
Heun-Johnson et al. 2019	12521	5518766	0.00227 (0.00223, 0.00231)	1.21
Skoglund et al. 2019	940	384103	0.00245 (0.00230, 0.00261)	1.21
Chen et al. 2023	1892	584958	0.00323 (0.00309, 0.00338)	1.21
Meinhofer et al. 2022	87181	20914591	0.00417 (0.00414, 0.00420)	1.21
Hesselman et al. 2020	1650	377381	0.00437 (0.00417, 0.00459)	1.21
Shen et al. 2021	6509	1463302	0.00445 (0.00434, 0.00456)	1.21
Lee et al. 2022	175	37641	0.00465 (0.00401, 0.00539)	1.20
Dindo et al. 2017	47	5404	0.00870 (0.00655, 0.01155)	1.14
Khan et al. 2018	5	517	0.00967 (0.00414, 0.02244)	0.76
Bjorkstedt et al. 2022	64	6189	0.01034 (0.00811, 0.01318)	1.15
Hulsbosch et al. 2023	27	1682	0.01605 (0.01106, 0.02325)	1.02
Ehrenthal et al. 2024	3815	216684	0.01761 (0.01706, 0.01817)	1.21
Ma et al. 2022	28	1583	0.01769 (0.01227, 0.02545)	1.01
Lange et al. 2015	444	22962	0.01934 (0.01763, 0.02120)	1.19
Metz et al. 2024	218	9257	0.02355 (0.02065, 0.02684)	1.17
David et al. 2023	89	3373	0.02639 (0.02149, 0.03236)	1.11
Obrochta et al. 2020	10	288	0.03472 (0.01897, 0.06273)	0.59
Abdus-salam et al. 2022	15	380	0.03947 (0.02407, 0.06410)	0.67
Conner et al. 2015	395	8138	0.04854 (0.04408, 0.05342)	1.17
Ayers et al. 2024	16	303	0.05281 (0.03276, 0.08405)	0.60
Li et al. 2024	53	887	0.05975 (0.04597, 0.07733)	0.90
Cena et al. 2021	64	934	0.06852 (0.05402, 0.08656)	0.91
Nath et al. 2018	39	545	0.07156 (0.05279, 0.09633)	0.78
Coleman-Cowger et al. 2018	45	500	0.09000 (0.06794, 0.11831)	0.75
Dikmen-Yildiz et al. 2017	104	950	0.10947 (0.09117, 0.13092)	0.92
Van Heyningen et al. 2017	45	376	0.11968 (0.09066, 0.15640)	0.67
Mateus et al. 2022	504	3326	0.15153 (0.13975, 0.16412)	1.11
Zhang et al. 2025	477	3053	0.15624 (0.14379, 0.16955)	1.10
Premji et al. 2020	64	282	0.22695 (0.18192, 0.27932)	0.58
González-Mesa et al. 2020	138	514	0.26848 (0.23199, 0.30841)	0.76
Liu et al. 2024	289	1049	0.27550 (0.24932, 0.30332)	0.94
Subtotal (I^2 = 99.98013%, p = 0.00000)			0.02350 (0.02079, 0.02637)	39.12
with estimated predictive interval			. (0.00981, 0.04274)	
Heterogeneity between groups: p = 0.000				
Overall (I^2 = 99.99131%, p = 0.00000);			0.01900 (0.01734, 0.02074)	100.00
with estimated predictive interval			. (0.00643, 0.03790)	

-.2 0 .2 .4

Supplemental Table 6: Meta-regression

	Coefficient	Standard error	p-value	95% CI
**Region:**				
Africa (Reference)	-	-	-	-
Asia	0.0166129	0.0234714	0.481	[-0.030 - 0.062]
Europe	-0.0446205	0.0213822	0.040	[-0.087 - -0.002]
North America	-0.0477656	0.0206808	0.023	[-0.089 - -0.007]
Oceania	-0.0287395	0.0306062	0.350	[-0.896 - 0.032]
South America	0.0030323	0.0279591	0.914	[-0.053 - 0.059]
**Ascertainment:**				
Objective (Reference)	-	-	-	-
Self-reported	0.0543562	0.009502	0.000	[0.035 - 0.073]
**Study design:**				
Cohort study (Reference)	-	-	-	-
Cross-sectional study	0.0198014	0.0108383	0.071	[-0.002 - 0.041]
**Depression:**				
Depression absent (Reference)	-	-	-	-
Depression present	0.0179557	0.0103797	0.087	[-0.003 - 0.039]
**World Bank income region:**				
High income (Reference)	-	-	-	-
Upper-middle income	0.0598767	0.0121134	0.000	[0.036 - 0.084]
Low-middle income	0.106385	0.0497032	0.035	[0.008 - 0.205]
Low income	0.0876082	0.0302133	0.005	[0.028 - 0.148]
**Number of conditions:**				
2 (Reference)	-	-	-	-
3	-0.0164264	0.015152	0.281	[-0.047 - 0.014]
**Mental health only:**				
Not mental health only (Reference)	-	-	-	-
Mental health only	0.0129764	0.0108146	0.233	[-0.009 - 0.034]

Supplemental Figure 7: Sensitivity analysis excluding 10 lowest quality studies

Author name and year of publication	Number of pregnant women with most common multimorbidity	Total number of participants	ES (95% CI)	% Weight
Mogos et al. 2023	95	19754535	0.00000 (0.00000, 0.00001)	1.33
Kishore et al. 2019	1439	42317648	0.00003 (0.00003, 0.00004)	1.33
Potnuru et al. 2024	775	18161315	0.00004 (0.00004, 0.00005)	1.33
Wu et al. 2020	259	4240284	0.00006 (0.00005, 0.00007)	1.33
Nasiri et al. 2021	997	13792544	0.00007 (0.00007, 0.00008)	1.33
Magtanong et al. 2019	1144	12592676	0.00009 (0.00009, 0.00010)	1.33
Bourjeily et al. 2017	176	1577632	0.00011 (0.00010, 0.00013)	1.33
Lopez-de-Andrés et al. 2024	225	1995953	0.00011 (0.00010, 0.00013)	1.33
Akaishi et al. 2023	94	804617	0.00012 (0.00010, 0.00014)	1.33
Fabre et al. 2021	447	3667461	0.00012 (0.00011, 0.00013)	1.33
Mogos et al. 2019	13090	58679098	0.00022 (0.00022, 0.00023)	1.33
Margolese et al. 2025	3435	9096788	0.00038 (0.00037, 0.00039)	1.33
Ren et al. 2021	377	937422	0.00040 (0.00036, 0.00044)	1.33
Kendle et al. 2022	21186	46975745	0.00045 (0.00044, 0.00046)	1.33
Vigod et al. 2019	371	802629	0.00046 (0.00042, 0.00051)	1.33
Valinder et al. 2023	706	1220822	0.00058 (0.00054, 0.00062)	1.33
Kassee et al. 2023	791	1128776	0.00070 (0.00065, 0.00075)	1.33
Chen et al. 2023	57263	70038267	0.00082 (0.00081, 0.00082)	1.33
Männistö et al. 2016	251	223394	0.00112 (0.00099, 0.00127)	1.33
Ferrara et al. 2022	60	43886	0.00137 (0.00106, 0.00176)	1.32
Larsen et al. 2016	130	83731	0.00155 (0.00131, 0.00184)	1.33
Raina et al. 2021	15042	9097355	0.00165 (0.00163, 0.00168)	1.33
Bjørk et al. 2015	189	107516	0.00176 (0.00152, 0.00203)	1.33
Huang et al. 2023	983	539950	0.00182 (0.00171, 0.00194)	1.33
Edvardsson et al. 2022	1151	595792	0.00193 (0.00182, 0.00205)	1.33
Iacobelli et al. 2017	125	61062	0.00205 (0.00172, 0.00244)	1.32
Heun-Johnson et al. 2019	12521	5518766	0.00227 (0.00223, 0.00231)	1.33
Skoglund et al. 2019	940	384103	0.00245 (0.00230, 0.00261)	1.33
Chen et al. 2023	1892	584958	0.00323 (0.00309, 0.00338)	1.33
Zahid et al. 2024	9078	2371661	0.00383 (0.00375, 0.00391)	1.33
Meinhofer et al. 2022	87181	20914591	0.00417 (0.00414, 0.00420)	1.33
Hesselman et al. 2020	1650	377381	0.00437 (0.00417, 0.00459)	1.33
Shen et al. 2021	6509	1463302	0.00445 (0.00434, 0.00456)	1.33
Lee et al. 2022	175	37641	0.00465 (0.00401, 0.00539)	1.32
Chen et al. 2017	8	1438	0.00556 (0.00282, 0.01094)	1.10
Houtchens et al. 2018	267	41553	0.00643 (0.00570, 0.00724)	1.32
Litman et al. 2022	331	43612	0.00759 (0.00682, 0.00845)	1.32
Salahuddin et al. 2020	12467	1434441	0.00869 (0.00854, 0.00884)	1.33
Dindo et al. 2017	47	5404	0.00870 (0.00655, 0.01155)	1.26
Orós et al. 2023	159	16803	0.00946 (0.00811, 0.01104)	1.31
Khan et al. 2018	5	517	0.00967 (0.00414, 0.02244)	0.84
Bjorkstedt et al. 2022	64	6189	0.01034 (0.00811, 0.01318)	1.27
Shuffrey et al. 2022	63	5822	0.01082 (0.00847, 0.01382)	1.26
Welander et al. 2021	62	4831	0.01283 (0.01002, 0.01642)	1.25
Maack et al. 2019	59	3965	0.01488 (0.01155, 0.01915)	1.23
Hulsbosch et al. 2023	27	1682	0.01605 (0.01106, 0.02325)	1.13
Hartwell et al. 2023	2285	140817	0.01623 (0.01558, 0.01690)	1.33
Ehrenthal et al. 2024	3815	216684	0.01761 (0.01706, 0.01817)	1.33
Ma et al. 2022	28	1583	0.01769 (0.01227, 0.02545)	1.11
Lange et al. 2015	444	22962	0.01934 (0.01763, 0.02120)	1.31
Yang et al. 2016	64	2899	0.02208 (0.01733, 0.02809)	1.20
Metz et al. 2024	218	9257	0.02355 (0.02065, 0.02684)	1.29
Brown et al. 2024	51181	2014508	0.02541 (0.02519, 0.02562)	1.33
Ertmann et al. 2022	34	1290	0.02636 (0.01892, 0.03660)	1.07
David et al. 2023	89	3373	0.02639 (0.02149, 0.03236)	1.22
Chhabria et al. 2024	10091	372895	0.02706 (0.02655, 0.02759)	1.33
Laine et al. 2018	215	7750	0.02774 (0.02431, 0.03164)	1.28
Obrochta et al. 2020	10	288	0.03472 (0.01897, 0.06273)	0.64
Kemppinen et al. 2022	46	1273	0.03614 (0.02720, 0.04786)	1.07
Abdus-salam et al. 2022	15	380	0.03947 (0.02407, 0.06410)	0.74
Stanhope et al. 2022	595	14225	0.04183 (0.03866, 0.04524)	1.30
Girchenko et al. 2018	138	3117	0.04427 (0.03760, 0.05207)	1.21
Leal et al. 2020	200	4262	0.04693 (0.04097, 0.05369)	1.24
Conner et al. 2015	395	8138	0.04854 (0.04408, 0.05342)	1.28
López-de-Andrés et al. 2020	122050	2481479	0.04918 (0.04892, 0.04945)	1.33
Ayers et al. 2024	16	303	0.05281 (0.03276, 0.08405)	0.66
Friedman et al. 2016	180	3323	0.05417 (0.04697, 0.06239)	1.22
Belsti et al. 2024	3064	48502	0.06317 (0.06104, 0.06537)	1.32
Kattini et al. 2020	135	2073	0.06512 (0.05529, 0.07657)	1.16
Uguz et al. 2019	78	1154	0.06759 (0.05449, 0.08356)	1.05
Cena et al. 2021	64	934	0.06852 (0.05402, 0.08656)	1.00
Nath et al. 2018	39	545	0.07156 (0.05279, 0.09633)	0.85
Dalton et al. 2023	5815	75768	0.07675 (0.07487, 0.07866)	1.32
Coleman-Cowger et al. 2018	45	500	0.09000 (0.06794, 0.11831)	0.82
Pampaka et al. 2018	209	1912	0.10931 (0.09610, 0.12409)	1.15
Dikmen-Yildiz et al. 2017	104	950	0.10947 (0.09117, 0.13092)	1.01
Orta et al. 2015	146	1321	0.11052 (0.09473, 0.12857)	1.08
Van Heyningen et al. 2017	45	376	0.11968 (0.09066, 0.15640)	0.73
Bitew et al. 2019	180	1311	0.13730 (0.11973, 0.15699)	1.08
Zhang et al. 2025	477	3053	0.15624 (0.14379, 0.16955)	1.21
David et al. 2023	205	1279	0.16028 (0.14120, 0.18140)	1.07
Alnasheet et al. 2024	85	513	0.16569 (0.13603, 0.20033)	0.83
Overall (I^2 = 99.99218%, p = 0.00000)			0.01496 (0.01342, 0.01658)	100.00
with estimated predictive interval			. (0.00418, 0.03209)	

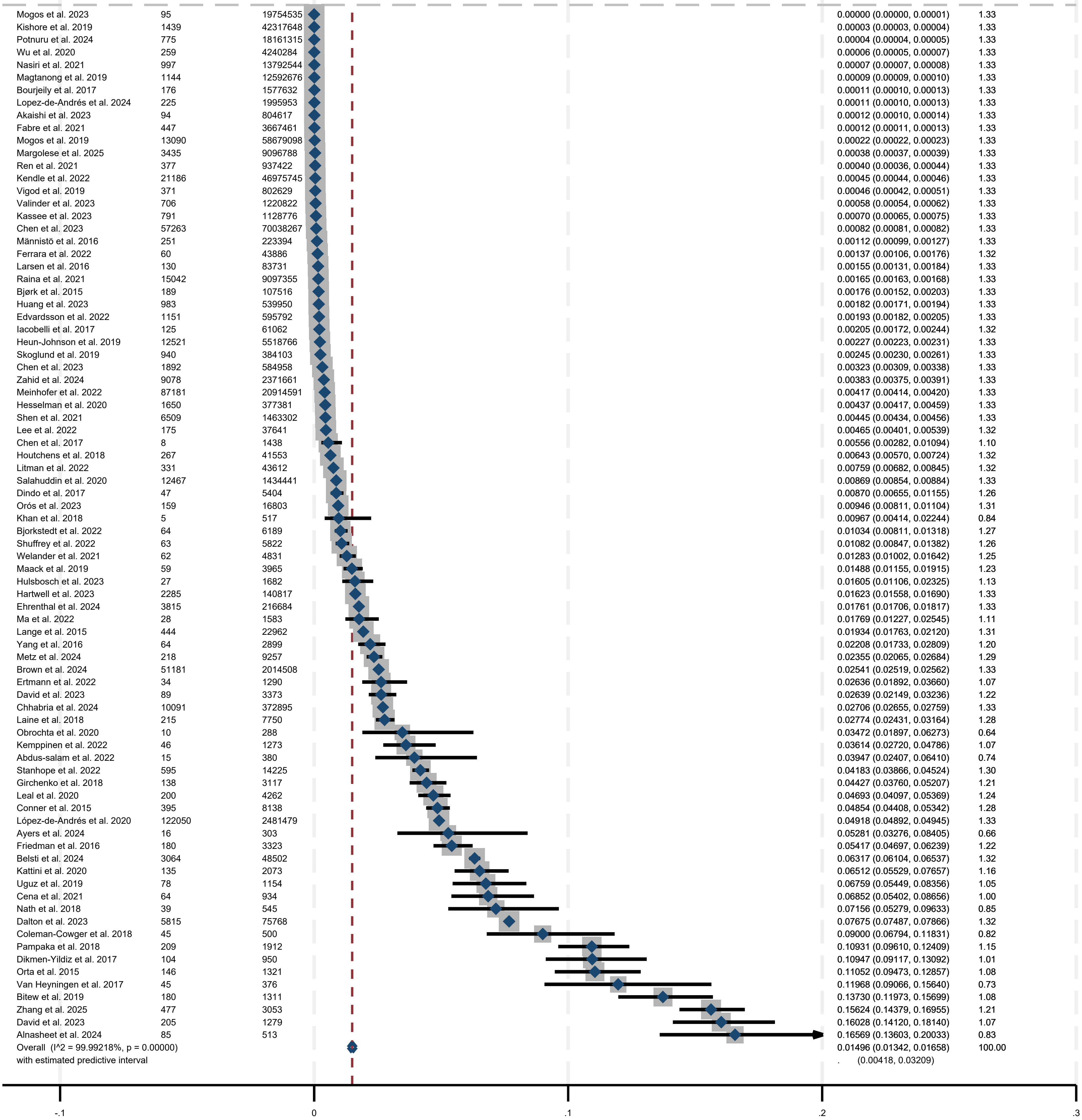

Supplement: Supplementary file 1 — Supplementary Material 1. [file 12889_2026_26545_MOESM1_ESM.pdf]
